# Comparative Analysis of Codon Optimization Tools: Advancing toward a Multi-Criteria Framework for Synthetic Gene Design

**DOI:** 10.4014/jmb.2411.11066

**Published:** 2025-04-10

**Authors:** Eden A. Demissie, Seo-Young Park, Je Hun Moon, Dong-Yup Lee

**Affiliations:** 1 School of Chemical Engineering, Sungkyunkwan University, Suwon 16419, Republic of Korea; 2 School of Medicine, Kyungpook National University, Daegu 41944, Republic of Korea

**Keywords:** Codon optimization, synthetic biology, recombinant protein production, translational efficiency, host-specific expression

## Abstract

Codon optimization is an essential technique in synthetic biology and biopharmaceutical production, enhancing recombinant protein expression by fine-tuning genetic sequences to match the translational machinery and codon usage preferences of specific host organisms. This study presents a comprehensive comparative analysis of widely used codon optimization tools, focusing on their capacity to reflect host-specific codon biases, design principles, and parameters. Industrially relevant target proteins were evaluated in *Escherichia coli*, *Saccharomyces cerevisiae*, and CHO cells, uncovering significant variability in sequence design and clustering patterns across tools. Tools such as JCat, OPTIMIZER, ATGme, and GeneOptimizer demonstrated strong alignment with genome-wide and highly expressed gene-level codon usage, achieving high codon adaptation index (CAI) values and efficient codon-pair utilization. Conversely, tools like TISIGNER and IDT employed different optimization strategies that frequently produced divergent results. Other key parameters, including GC content, mRNA secondary structure stability (ΔG), and codon-pair bias (CPB), were analyzed to elucidate their influence on translational efficiency. While increased GC content enhanced mRNA stability in *E. coli*, A/T-rich codons in *S. cerevisiae* minimized secondary structure formation, and moderate GC content in CHO cells balanced mRNA stability and translation efficiency. Our findings highlight the limitations of single-metric approaches and advocate for a multi-criteria framework that integrates CAI, GC content, mRNA folding energy, and codon-pair considerations. This integrative strategy enables the design of tailored genetic sequences that meet host-specific requirements, advancing synthetic gene design for biotechnological innovation and precision biopharmaceutical applications.

## Introduction

Recombinant protein production is a cornerstone of modern biotechnology, underpinning advancements in biopharmaceuticals, industrial enzymes, and agricultural innovations. Recent advancements in *de novo* gene synthesis technologies have enabled the design and synthesis of genes tailored for the efficient production of recombinant proteins that are more accessible, cost-effective, and scalable [1-3]. A key requirement for successful recombinant protein production is the development of robust and stable host organisms capable of expressing high-quality proteins in large quantities. Commonly used host systems for this purpose include microbial and mammalian cells, particularly *Escherichia coli*, *Saccharomyces cerevisiae*, and Chinese hamster ovary cells. These hosts are widely used for producing enzymes, pharmaceuticals, and chemicals, with many biopharmaceuticals approved by regulatory bodies like the FDA and EMA being produced in *E. coli*, *S. cerevisiae*, and mammalian cells over recent decades [4, 5].

One of the main challenges in expressing heterologous proteins in these systems is the difficulty of achieving high expression levels outside of the genés native context. Gene sequences that encode a protein in one organism may not be efficiently translated into another, primarily due to differences in codon usage. This phenomenon, known as codon usage bias, affects translation rates and can significantly impact the economics of recombinant protein production [6-8]. Codon optimization, a strategy to align the codon usage of a target gene with the preferred codons of the host organism, has emerged as an effective solution to overcome these challenges [9-11].

Codon optimization leverages the degeneracy of the genetic code, which allows multiple synonymous codons to encode the same amino acid. By modifying the codon sequence to align with the host’s codon preference, codon optimization enhances translational efficiency and protein yield [12, 13]. For example, codon optimization has successfully improved the production of synthetic biofuels in *S. cerevisiae* and *Clostridium thermocellum* [14-16], therapeutic proteins in *E. coli* [17], and vaccines for human use [18-21].

The optimization process typically begins by analyzing the target protein sequence and the host organism’s codon usage bias, often derived from highly expressed genes within the host’s genome or transcriptome data. Design criteria/parameters such as the Codon Adaptation Index (CAI), Individual Codon Usage (ICU), Codon Context (CC), GC content, and mRNA secondary structure stability and constraints such as restriction enzyme site and repeats are considered to guide and control the optimization, thereby delivering optimized sequences [22-24] and ensure efficient translation and high expression levels of the target protein. Several codon optimization tools have been developed to streamline this process, providing accessible platforms for gene design and optimization (Supplementary Information). However, inconsistencies between tools, due to a lack of universal metrics and standardized design criteria, can lead to variations in optimized sequences, which in turn affects recombinant protein activity and yield [4, 25]. Without a consistent framework for defining an “optimized” sequence, each tool’s results may differ, complicating the synthetic gene design.

This study shifts the focus from merely comparing codon optimization tools to highlighting the importance of incorporating multiple design criteria into a holistic optimization approach. We emphasize the significance of key design criteria/parameters such as CAI, GC content, mRNA secondary structure, and CC in achieving effective codon optimization. By conducting case studies in *E. coli*, *S. cerevisiae*, and CHO cells, we illustrate the necessity of a tailored approach that addresses each host system’s unique biological and technical requirements.

## Materials and Methods

### Selection of Codon Optimization Tools

We examined a range of available codon optimization tools to ensure robust coverage and functionality across different optimization criteria. Tools were carefully reviewed for accessibility, species specificity, and functionality, as such selecting ten widely used tools: JCat [26], OPTIMIZER [27], ATGme [28], TISIGNER [29], GenSmart [30], ExpOptimizer [31], IDT [32], Genewiz [33], GeneOptimizer [34], and Vector Builder [35]. Each tool was set to its default settings for consistency unless otherwise specified, ensuring a standardized basis for comparison. These tools differ in their level of sophistication and range of parameters (Table 1). Key parameters integrated into most of these tools include the CAI, mRNA secondary structure, GC content, and restriction enzyme avoidance, with specific functionalities varying between tools. We compared tools side-by-side using these parameters, with the aim of identifying the specific effects of each on codon optimization outcomes.

### Target Genes and Host Strains

To provide a representative basis for analysis, we selected three target proteins with varying lengths and industrial relevance: human insulin (INS, Accession Number - NM_001185098.2), α-amylase (T310_7718, Accession Number - XM_013469492.1), and Adalimumab (Humira) heavy and light chains (Accession Numbers - LQ506328.1 and LQ506329.1, respectively) (Table 2). These sequences were sourced from the National Center for Biotechnology Information (NCBI) database. The selected host organisms, *E. coli* (K12 strain), *S. cerevisiae* (S288C strain), and CHO (K1 strain), are widely used in recombinant protein production due to their well-characterized genomic data and industrial applicability. The restriction enzymes used were EcoRI, ApaI, and NcoI respectively. Each gene was optimized using the ten selected tools for expression in these hosts.

### Codon Bias and Design Parameters

To construct the host-specific codon bias, we used genome and transcriptome datasets from the Gene Expression Omnibus (GEO) repository: *E. coli* K12 (GSE263906), *S. cerevisiae* S288C (GSE208095), and CHO K1 (GSE75521) [5]. These datasets cover recent years, from 2021 to 2024, except for CHO data from 2019, due to data limitations. From these datasets, genes in the top 10% for *E. coli* and *S. cerevisiae* and top 5% for CHO (due large genome) were used as references to compute codon usage bias of both the genome and highly expressed genes, considering only coding sequences that start with “ATG”. Codon frequencies were calculated to establish the host’s codon usage bias, and the CAI was computed for each optimized sequence to measure the alignment with host-specific preferences [36].

CAI is calculated using the following equation:


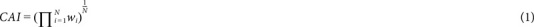


where N is the total number of codons in the sequence, and wi is the relative adaptiveness of each codon computed as:


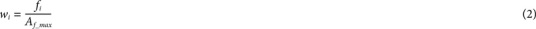


where *f_i_* and *A_f_max_* are the number of occurrences of synonymous codons and the number of occurrences for the most frequently used codon in the amino acid of codon *i*, respectively.

In addition to CAI, other key design parameters were analyzed to assess optimization performance. Calculated as the percentage of guanine and cytosine in a sequence, GC content impacts mRNA stability and translation efficiency, with optimal ranges varying between organisms. For each optimized sequence, GC content was compared to the host organism’s typical GC composition. The secondary structure of mRNA was evaluated by calculating Gibbs free energy (ΔG), a key indicator of structural stability [37-39]. The minimum folding energy (MFE) was predicted using RNAFold [38], which estimates the most stable energetic configuration of mRNA molecules by computing base pair probabilities and circular RNA folding. This analysis provides insights into the mRNA stability by assessing changes in Gibbs free energy during the folding process. To ensure a comprehensive analysis, Gibbs free energy was also calculated using UNAFold [40] and RNAstructure [41], complementing the values obtained from RNAFold. For further analysis, ICU and CC fitness were analyzed by calculating codon and codon-pair frequencies and scoring them based on the negative Manhattan distance between host and optimized sequence distributions [24, 42]. Codon-pair bias (CPB) [43, 44], calculated as the mean score for all codon pairs in a sequence, is an additional measure to ensure compatibility with host translation machinery.

ICU is evaluated as follows:


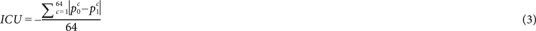


where $p_{0}^{c}$ and $p_{1}^{c}$ are the normalized codon frequencies for each codon in the host and target sequences, respectively and can be computed with the following general equation:


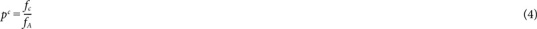


where *f_c_* and *f_A_* are the codon occurrence count and the count of occurrence of the amino acid.

CC can be computed as follows:


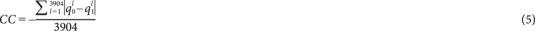


where $q_{0}^{l}$ and $q_{1}^{l}$ are normalized frequency values of each codon pair for the host and target sequences, respectively, and calculated as:


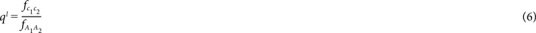


where *f*_*c*_1___*c*_2__ and *f*_*A*_1___*A*_2__ are codon pair and amino acid pair occurrence counts.

### Data Analysis and Statistical Tools

All statistical analyses and data visualizations were conducted using GraphPad Prism (version 10) and OriginPro (version 10). We used Principal Component Analysis (PCA) to evaluate clustering patterns based on codon usage and checked other indices such as CAI (either highly expressed genes or genome) across different optimization tools to verify the clusters, providing insights into tool-specific biases and highlighting distinct optimization approaches. The PCA allowed us to visualize the grouping of tools by the similarity of codon usage profiles, GC content, and mRNA structure, facilitating comparisons of the functional differences between tools.

## Results and Discussion

### Host-specific Codon Bias and Sequence Optimization

**Fig. 1:**
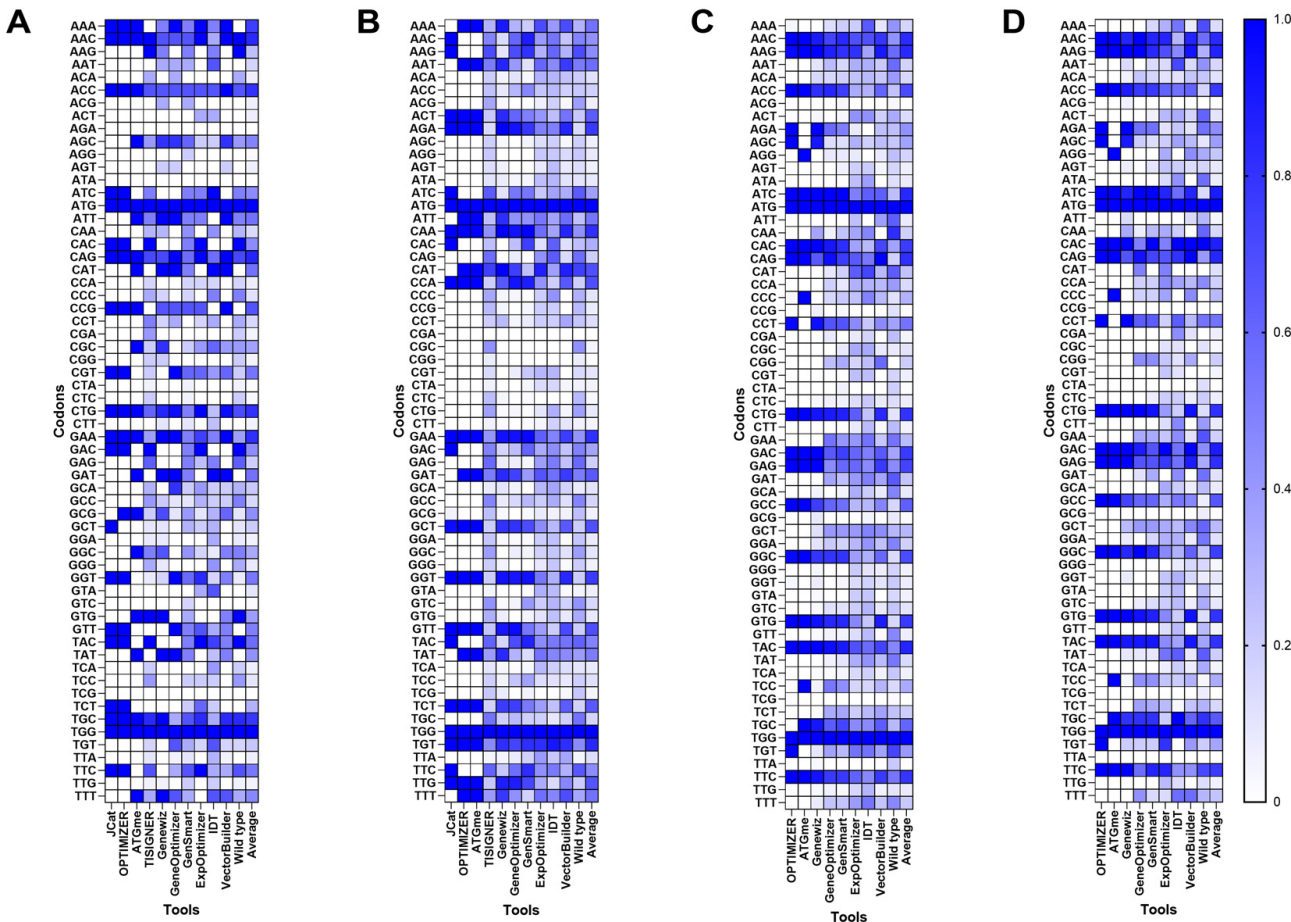
Codon usage frequencies of optimized sequences by tools. The distribution was determined by the codon usage frequencies, *p^c^*, of synonymous codons across the tools using heatmap. (**A**) Insulin in *E. coli*, (**B**) α-Amylase in *S. cerevisiae*, (**C**) Humira (heavy chain) in CHO cells, and (**D**) Humira (light chain) in CHO cells.

The distribution of optimized sequences is strongly influenced by host-specific codon biases, which vary significantly among organisms. To investigate this, we analyzed industrially relevant target proteins optimized for different host organisms using a variety of codon optimization tools, each of which incorporates distinct algorithms and codon usage preferences. Given the degeneracy of the genetic code, multiple synonymous sequences can encode the same protein, allowing infinite combinations of mRNA sequences for a single protein, resulting in variability among the optimized sequences produced by different tools (Supplementary Information). The codon distribution of optimized sequences (Fig. 1) indicated that tools such as JCat, OPTIMIZER, and ATGme adopted a ‘one amino acid-one codon’ strategy by selecting a single codon per amino acid to maximize CAI value (0 ≤ CAI ≤ 1, where 1 represents the maximum) in general.

**Fig. 2:**
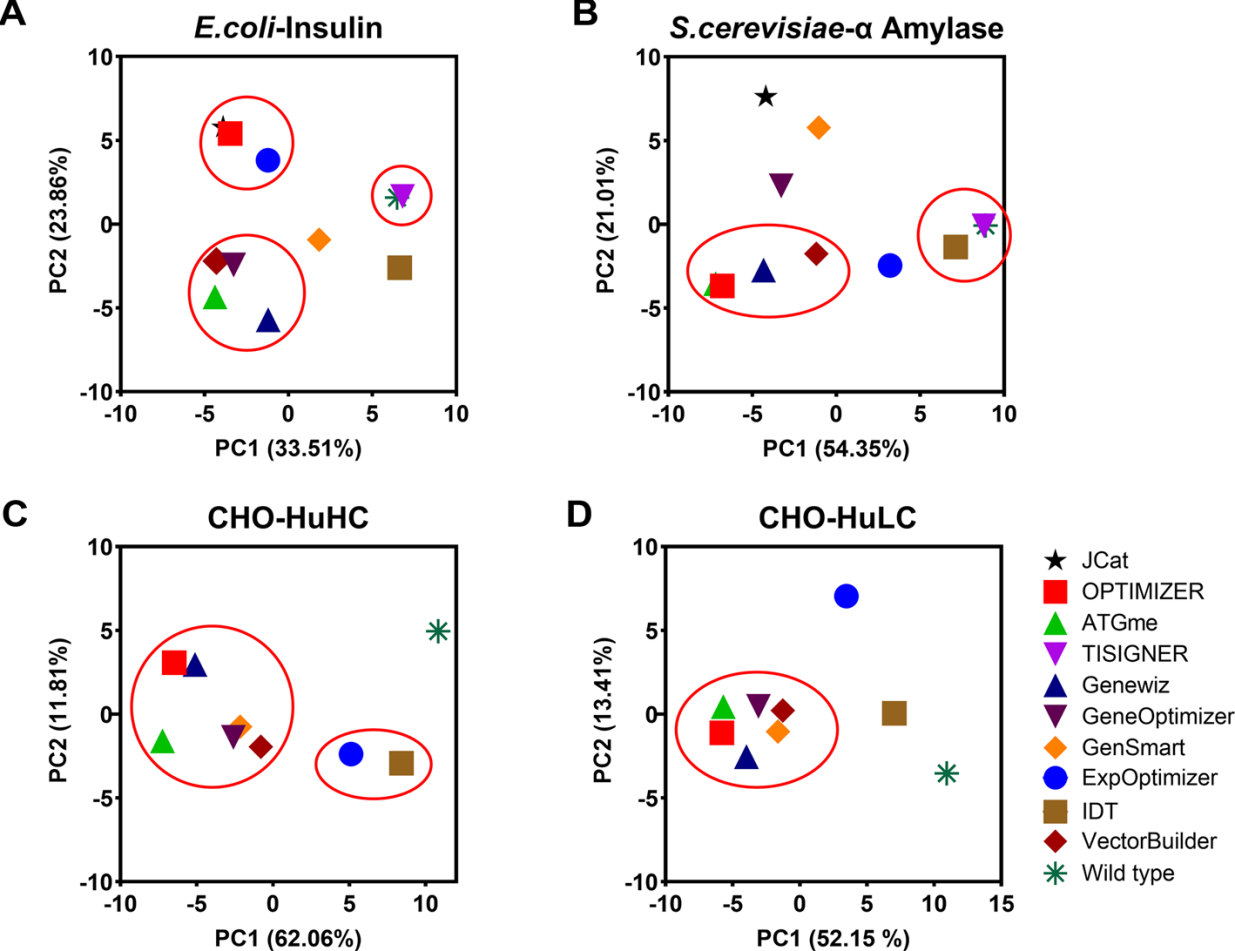
PCA plots for codon optimized sequences designed by tools. (**A**) Insulin in *E. coli*, (**B**) α-Amylase in *S. cerevisiae*, (**C**) Humira (heavy chain) in CHO cells, and (**D**) Humira (light chain) in CHO cells in accordance with their cluster.

**Fig. 3:**
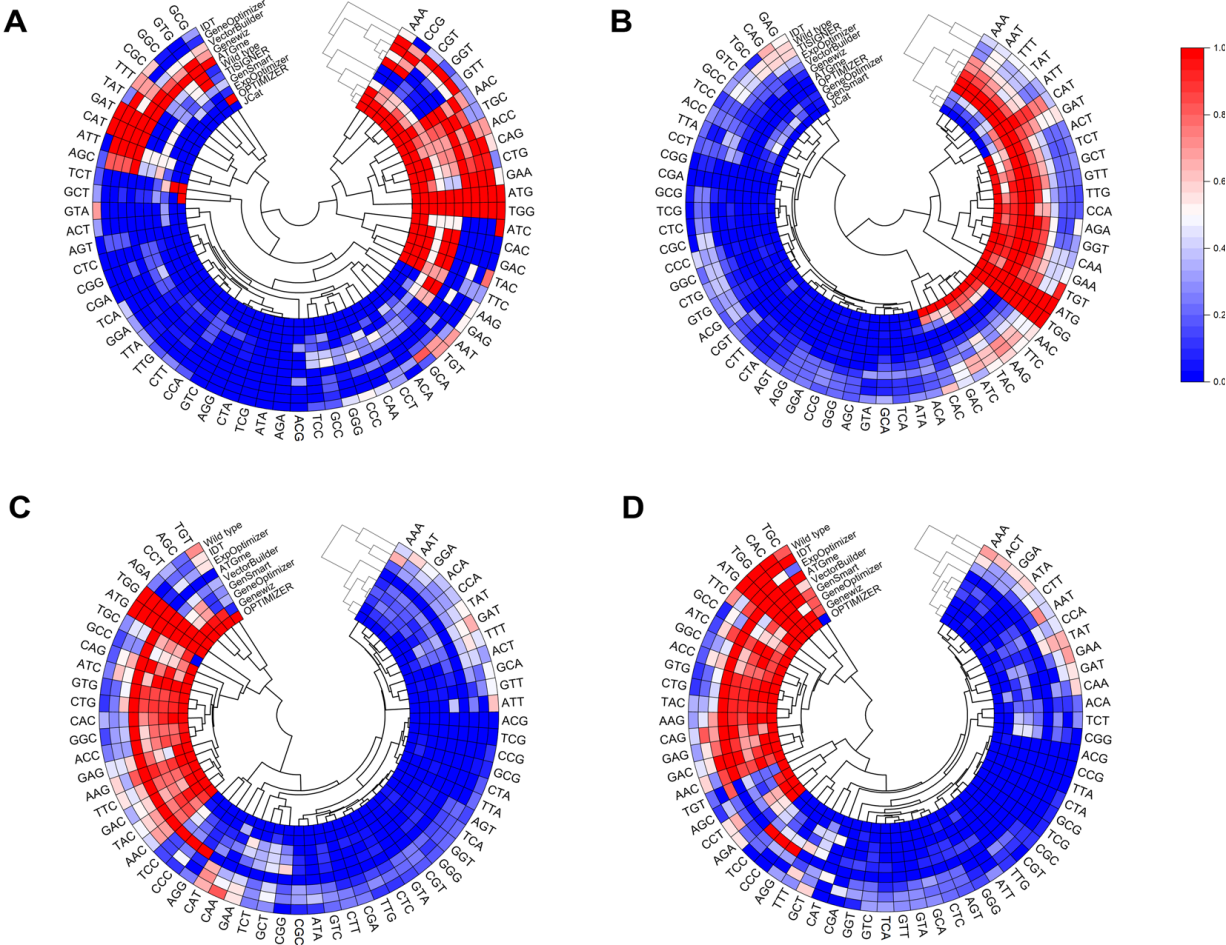
Hierarchical clustering of codon optimized sequences designed by tools. The dendrogram analysis depicts the hierarchical tree-like clustering of tools according to their unique codons in (**A**) Insulin, (**B**) α-Amylase, (**C**) Humira (heavy chain), and (**D**) Humira (light chain).

Optimized sequences in *E. coli* exhibited a clear preference for codons comprising CTG, GAA, GGT, and CAG while codons such as AGA, ATA, and TCG were rarely or never used. Interestingly, PCA identified three distinct clusters (Fig. 2A): cluster 1 (JCat, OPTIMIZER, and ExpOptimizer), cluster 2 (ATGme, Genewiz, GeneOptimizer, and VectorBuilder), and cluster 3 (TISIGNER and Wild type). The tools for cluster 1 showed high CAI values for both genome-wide and highly expressed (HE) gene-level biases, indicating a preference for codons dominant in both datasets. Key codons in this cluster included CTG, CAG, CCG, GTT, GAA, TAC, and CAC for both levels, with additional codons GCT, TTC, GGT, CGT, and TCT at the HE level contributing to the elevated CAI value (Fig. 3A). In cluster 2, codons such as CGC and AAT were predominantly used, aligning with genome-wide biases while cluster 3 group displayed minimal optimization, with codon usage closely resembling wild-type sequences. Codons such as CTG, AAC, TGC, and CAG were shared across all clusters, highlighting their universal role in enhancing protein expression. These findings are consistent with previous studies demonstrating the association of frequently used codons, such as CAG (Glutamine) and GCT (Alanine), in highly expressed *E. coli* genes with improved protein expression efficiency [45]. The optimization of the α-amylase gene (622 amino acid) in *S. cerevisiae* revealed a distinct preference for A/T-rich codons. Frequently used codons included TCT, ACT, GGT, TTG, GTT, GAT, and GCT each appearing more than 25 times on average, where CGA was absent across all tools. The PCA analysis (Fig. 2B) identified two clusters: cluster 1 (OPTIMIZER, ATGme, Genewiz, and VectorBuilder) and cluster 2 (TISIGNER, IDT, and Wild type). The tools for cluster 1 achieved higher CAI values and favored codons such as GAT, GGT, ATT, TTG at both genome-wide and HE levels while AAT and TAT were prevalent at the genome level (Fig. 3B). In cluster 2, limited optimization was observed, with codons TGT, GAT, and AAT used at both levels, often reflecting less favorable codon usage and resulting in suboptimal outcomes. The observed variation in codon preferences highlights the need for tools tailored to the unique genomic and translational landscapes of *S. cerevisiae*.

For CHO cells, we analyzed the heavy chain (HC, 422 amino acids) and light chain (LC, 215 amino acids) of Adalimumab. Due to limited tool support for CHO cells, JCat and TISIGNER were excluded from the analysis. Note that wild type sequences initially exhibited the highest CAI values at the genome level, but their performance decreased significantly when HE gene data were considered (Supplementary Information). PCA (Fig. 2C) revealed two major clusters for HC optimization. Cluster 1 (OPTIMIZER, ATGme, Genewiz, GeneOptimizer, GenSmart, and VectorBuilder) frequently used codons such as GCC, GAC, GAG, TTC, GGC, CAC, ATC, AAG, CTG, and CAG (Fig. 3C) whereas tools for cluster 2 (ExpOptimizer and IDT) favored codons like GCT, TGT, GAC, and GAG, reflecting distinct optimization strategies. For LC optimization, a single cluster (Fig. 2D) emerged across tools, with codon usage patterns closely mirroring HC Cluster 1 (Fig. 3D). Across both HC and LC, 18 codons were consistently favored at both genome-wide and HE levels, supporting their role for efficient protein translation.

Our findings indicate consistent clustering of tools such as OPTIMIZER, ATGme, VectorBuilder, GeneOptimizer, and Genewiz across host organisms, reflecting systematic alignment with host-specific codon usage biases. In contrast, tools such as ExpOptimizer, IDT, and TISIGNER displayed divergent codon preferences, often resulting in distinct optimization outcomes. These results underscore the importance of selecting codon optimization tools which design coding sequences based on the target host’s codon usage profile, design criteria (*e.g.*, CAI, ICU, CC, etc) and the specific parameter settings (*e.g.*, GC content, mRNA secondary structure, etc) for the enhanced expression of target proteins.

### GC Content Analysis

GC content is a crucial factor in codon optimization, as extremes in GC content can disrupt translation by promoting excessive mRNA secondary structure formation. The GC content of a sequence, represented as the percentage of guanine (G) and cytosine (C) bases, generally falls within a range of 30% to 70% to ensure proper transcription elongation and translation efficiency. For optimized sequences in this study, GC content ranged within this range, aligning closely with each host genome’s natural composition. This balance in GC content helps to prevent issues such as mRNA instability and ribosomal stalling, both of which can occur if GC content is too high or too low.

In *E. coli*, optimized sequences exhibited a GC content above 50%, with values typically ranging from 51% to 64%. This G/C richness is consistent with the GC-rich nature of the *E. coli* genome. Codons contributing to this elevated GC content included CTG, GGT, CAG, TGC, and GCG, which are commonly used in *E. coli*’s highly expressed genes [46]. For instance, Wild type insulin, used as a baseline for comparison, has a GC content of 64.56%, indicating that the optimized sequences retained a similar GC composition, enhancing compatibility with the host’s translational machinery.

Optimized sequences for *S. cerevisiae* showed a lower GC content, ranging from 32% to 46% (except TISIGNER), reflecting the organism's preference for A/T-rich sequences [46, 47]. This A/T bias minimizes mRNA secondary structure formation and conforms to *S. cerevisiae*’s genomic profile, and will help enhance transcription and translation efficiency [48]. Frequently used codons, such as TCT, ACT, TTG, GGT, and GAT, contributed to the observed A/T richness. The Wild type sequence, with a GC content of 52.38%, had a higher GC content than the optimized sequences, highlighting the tools' adaptation to the host's natural bias.

For CHO cells, widely used in biopharmaceutical production, GC content ranged from 48% to 66.08% for the heavy chain (HC) and 49% to 65% for the light chain (LC) of Adalimumab (Humira). This moderate GC enrichment supports mRNA stability while avoiding excessive secondary structure formation, particularly in longer sequences, ensuring balanced translation efficiency.

A positional analysis of GC and AT content across codon positions (first, second, and third) revealed distinct host-specific patterns. In *E. coli*, GC content followed the pattern GC1 > GC3 > GC2, with a preference for high GC at the first codon position, consistent with its overall GC-rich profile. For *S. cerevisiae*, the pattern was AT3 > AT1 > AT2, indicating a strong A/T bias at the third position, while the pattern (GC3 > GC1 > GC2) in CHO cells showed a preference for GC-rich codons at the third position, which supports mRNA stability and efficient translation. These position-specific fluctuations significantly influence mRNA stability and translation. High GC content at specific positions stabilizes mRNA but may slow translation due to increased secondary structure, while lower GC content reduces stability but accelerates translation. Tailoring GC content for each host's specific needs ensures compatibility with their natural biases and supports efficient protein expression.

### mRNA Secondary Structure and Folding Energy

mRNA secondary structure stability, often measured by folding energy (ΔG), computed through analysis of base pair probabilities and circular RNA folding, was recently recognized as one of critical parameters in codon optimization, as it potentially influences both translation initiation and elongation [48, 49]. More stable secondary structures, represented by higher absolute Gibbs free energy (more negative), can hinder ribosome binding and movement, thus reducing translation efficiency. Our analysis indicated that sequence length [50] and GC content significantly impacted mRNA folding energy, with longer and GC-rich sequences tending to form more stable, complex secondary structures. These structures can impose additional energetic costs, affecting the speed and accuracy of translation.

In *E. coli*, optimized sequences with higher GC content were associated with more stable mRNA structures, crucial for efficient translation in this host. The relationship between GC content and ΔG values showed that GC-rich sequences tend to have lower (more negative) ΔG, indicating greater stability. For example, optimized insulin sequences, with GC content ranging from 51% to 64%, exhibited ΔG values between -156 kcal/mol and -104 kcal/mol. This stability aligns well with *E. coli*'s translational requirements, as the host’s ribosomes are adapted to handle relatively stable structures.

For *S. cerevisiae*, though the influence of GC content on mRNA stability was less pronounced due to the generally lower GC composition of optimized sequences, a significant influence of sequence length had a considerable impact on overall ΔG values (Supplementary Information). Minor alterations in GC content resulted in substantial changes in ΔG values, for example, GC content ranging from 32%-46% had ΔG values between -556.10 kcal/mol and -397.7 kcal/mol reflecting a broader range, which we found contrary to *E. coli*. Despite the relatively higher stability, the A/T-rich composition of optimized sequences aligns with S. cerevisiaés natural codon usage bias, minimizing potential folding issues that might otherwise disrupt translation [48, 51].

In CHO cells, mRNA secondary structure played a particularly important role due to the longer lengths of sequences, *i.e.*, the heavy and light chains of Adalimumab. For these longer sequences, even small changes in GC content could significantly impact mRNA stability. In our analysis, the GC content for optimized heavy and light chains ranged from 48% to 66%, leading to ΔG values indicative of stable mRNA structures. This stability is necessary for efficient translation in CHO cells, especially given the host’s sensitivity to mRNA folding patterns at higher GC content levels. Thus, tools supporting the feature for secondary structure stability could suggest more efficient sequences in CHO cells.

To further understand the role of GC content in secondary structure formation, we examined changes in GC content (ΔGC) within sequences. The influence of ΔGC was more substantial in longer amino acid sequences, where even minor shifts in GC content resulted in notable impacts on mRNA folding energy and structure. G-C base pairs, being more stable than A-T pairs, contribute significantly to secondary structure stability [48, 51]. Sequences with higher GC content tend to form more stable secondary structures due to the stronger G-C base pairs, which involve three hydrogen bonds compared to the two in A-U pairs. An increase in GC content enhances the stability of secondary structures, such as hairpins, which are more frequently observed in GC-rich sequences [52], often causing ribosomes to pause, thereby affecting gene expression. Thus, sequences with higher GC content tend to form more robust structures, as seen in optimized Adalimumab heavy chain sequences and α-amylase. In addition to assessing ΔG, we evaluated whether restriction enzyme sites were present in optimized sequences. Restriction enzyme sites can affect cloning and expression strategies, but none were detected in the optimized sequences, indicating that all tools effectively avoided introducing these unwanted sites during optimization. The absence of these sites across optimized sequences enhances their suitability for downstream applications. Overall, our findings emphasize the importance of incorporating mRNA secondary structure stability into codon optimization, especially for longer sequences and hosts, *e.g.*, CHO cells that are sensitive to folding patterns. Tools that consider folding energy as a design parameter yield optimized sequences with improved stability, possibly facilitating efficient translation and enhancing protein expression although experimental verification is highly required.

### Design Criteria: Codon Usage and Pairing Parameters

We evaluated additional design parameters including ICU, CC fitness, and codon-pair bias (CPB). These metrics offer insights into how codon and codon-pair preferences impact protein expression across different hosts, complementing the GC content and CAI analyses discussed earlier. CC refers to the interaction between neighboring codons, while CPB measures the frequency of specific codon pairs and their compatibility with the host organism’s preferences. Both parameters have been identified as critical for translational efficiency [53]. ICU and CC fitness were computed using the negative Manhattan distance between the codon or codon pair distribution of the host and the target sequence. Values closer to 0 were considered optimal, indicating high similarity with the host’s codon usage pattern. Similarly, CPB quantifies how codon pairs align with the host’s translational machinery bias to provide an additional layer of sequence refinement, thus reflecting the degree of pair bias observed within the optimized sequence.

**Fig. 4:**
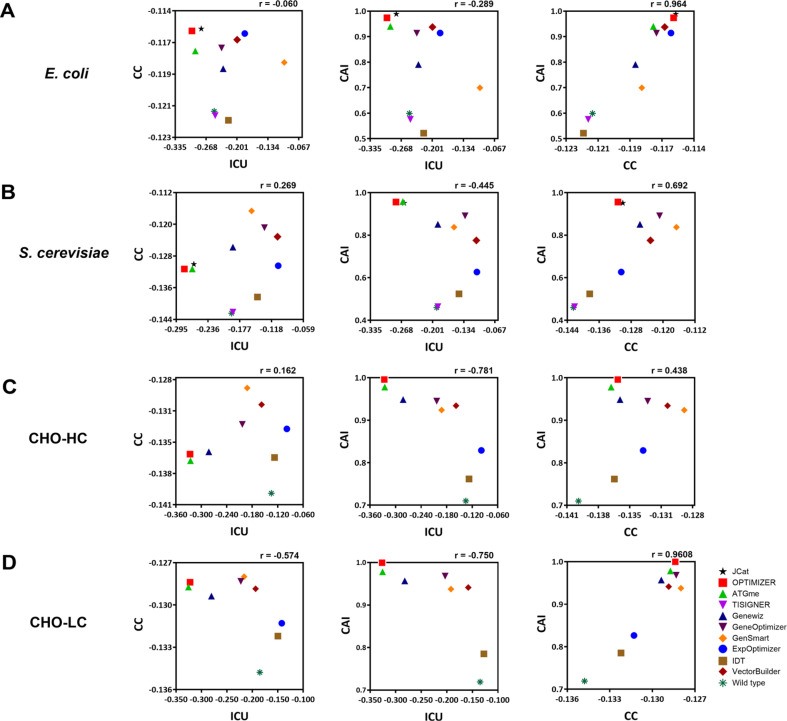
Correlation plot of three parameters (ICU, CC and CAI). Pearson correlation analysis was employed to investigate the interrelationships between the three parameters. (**A**) Insulin, (**B**) α-Amylase, (**C**) Humira (heavy chain), and (**D**) Humira (light chain). A moderate positive correlation was observed between CC and CAI in all four cases.

From the results, we analyzed the correlation between ICU, CC, and CAI, three design criteria in codon optimization. Interestingly, a moderate positive correlation was observed between CC and CAI (Fig. 4), which is attributable to codon usage constraints and sequence length. Shorter sequences, such as insulin and the light chain (LC) of Adalimumab, inherently limit amino acid and codon diversity, amplifying correlations as a result of the restricted codon pool. In contrast, longer sequences like α-amylase and the heavy chain (HC) displayed greater variability, which diluted the correlation between these metrics. In practice, highly expressed genes are characterized by both elevated CAI values and favorable codon pair distributions, suggesting that codon bias significantly influences in shaping a gene’s codon pair usage pattern. Despite claims by tools such as GenSmart to incorporate CC into their optimization algorithms, no significant improvement was observed compared to tools that did not explicitly include CC. This is in good agreement with previous studies suggesting a positive evolutionary correlation between codon usage and codon-pair distribution, indicating that these parameters can exert substantial effects on translation efficiency even without explicit integration [24, 54]. These findings emphasize that while CC and CAI are interrelated, their contributions to codon optimization are distinct, highlighting the significance of considering multiple metrics in tandem for effective codon optimization.

In *E. coli*, we identified specific CTG-based codon pairs such as CTGCTG and CTGTGG, which were prevalent across all clusters, emphasizing a strong preference for these pairs in highly expressed genes. In cluster analyses, cluster 1 and cluster 2 exhibited a tendency toward frequently used CTG pairs, while cluster 3 showed broader distribution patterns, including pairs such as AAGACC and ACCCGC (Supplementary Information). Previous studies have also reported that CTG is among the favored codons in *E. coli*, particularly in high-expression contexts, supporting our observations at both codon and codon-pair levels [55].

In *S. cerevisiae*, optimization favored A/T-rich codons such as ACT and TCT, which contribute to higher translational stability and, codon pairs including ACTACT, TCTTCT, and ACTGTT were commonly used, especially in cluster 1, while ACGGCC and TACAAC pairs were more prevalent in cluster 2. The consistent use of ACT, TCT, and their associated pairs aligns with *S. cerevisiae*’s preferences and underscores their role in stabilizing translational processes [14, 46]. These patterns reinforce the importance of tailoring codon-pair usage to the host's natural biases for optimized protein expression.

In CHO cells, codon pairs containing GTG, such as GTGGTG, GTGGAC, and GTGCTG, were prominent in the HC cluster 1 and LC sequences of Adalimumab. In contrast, pairs like ACTAAG and ACCGTG were more frequent in HC cluster 2. The preference for GTG-based pairs reflects CHO cells' affinity for valine-rich sequences, which are associated with improved stability and translation efficiency [54, 56]. These findings highlight the need to incorporate specific codon pair combinations into optimization workflows for CHO cells to address potential translational bottlenecks.

### Holistic Codon Optimization for Enhanced Protein Expression

Our study highlights the necessity of a comprehensive approach to codon optimization by integrating multiple design criteria selected with the specific requirements of the host organism. Codon usage preferences differ substantially across species, making reliance on a single metric (*e.g.*, CAI or GC content) insufficient to address the complexity of translational efficiency. While CAI and GC content provide valuable insights into general codon optimization, they do not encompass critical factors such as CC, mRNA folding stability, or codon-pair bias, all of which profoundly influence protein expression.

The analysis revealed significant variability in codon usage, GC content, and mRNA structure across different optimization tools. For example, CAI values fluctuated depending on the data source, with tools using highly expressed gene datasets often outperforming those relying on whole-genome data. However, tools focusing solely on CAI or GC content failed to account for other key parameters, such as mRNA secondary structure stability and codon-pair bias, which play crucial roles in translation efficiency, particularly for longer sequences such as those encoding therapeutic proteins like Adalimumab. These observations emphasize the inadequacy of a single-metric approach and the importance of adopting broader, multi-parameter optimization strategies.

Optimized sequences designed with additional criteria, such as mRNA secondary structure stability and codon-pair bias, can improve expression stability and translational efficiency. For instance, maintaining GC content within a host-specific optimal range prevents disruptions in transcription elongation and avoids excessive mRNA secondary structure formation, which can impede ribosomal progress. In addition, tools incorporating secondary structure considerations produced sequences that reduced ribosomal stalling, particularly in CHO cells, where fine-tuning these parameters is essential for achieving high protein yields [54]. In *E. coli*, sequences with high-frequency codon pairs, such as CTGCTG, demonstrated a strong potential for the enhanced translational efficiency, suggesting that codon-pair optimization, often overlooked in prokaryotic hosts, can significantly benefit protein expression even in robust translation systems [55].

The findings of this study advocate for a multi-faceted codon optimization framework that simultaneously considers multiple design criteria [57-61] including CAI, GC content, mRNA stability, and codon-pair bias. Each of these parameters plays a distinct yet interrelated role in optimizing translational efficiency. By tailoring these criteria to the biological and translational context of the host organism, researchers can maximize protein yield while minimizing potential bottlenecks. For example, CHO cells benefit from balanced GC content and codon-pair optimization to accommodate their sensitivity to mRNA stability, while *E. coli* thrives with high-frequency codon pairs that enhance translational speed and accuracy. *S. cerevisiae*, on the other hand, requires A/T-rich sequences aligned with its genomic bias to ensure efficient transcription and translation.

Tools like ExpOptimizer, IDT, and TISIGNER often exhibit distinct codon usage preferences, resulting in varying optimization outcomes. For example, TISIGNER exhibits a primary focus on enhancing translation initiation efficiency, whereas IDT places greater emphasis on achieving balanced codon usage to improve the overall translational efficiency of the gene. This highlights the importance of careful tool selection as optimization relies on considering the target host's codon usage profile, desired design criteria (*e.g.*, CAI, ICU, CC), and specific parameters (*e.g.*, GC content, mRNA secondary structure) to enhance protein expression. In addition, future advances in codon optimization tools, potentially those leveraging machine learning to adjust parameters based on extensive biological data [62], could improve accuracy and host compatibility. While some tools, such as ICOR and CHOExp, are available, their applicability still remains restricted due to the limited number of supported host organisms, as well as constraints in reliable training datasets and computation capability [63-65]. Regular updates to codon usage databases, like HIVE-CUT, KAZUSA and Codon Statistics, are also needed to keep pace with evolving genomic data [66-68]. Newer approaches, including deep learning, could further refine optimization by predicting codon patterns for specific hosts and applications [63, 69].

A holistic approach to codon optimization has far-reaching implications for biotechnology and biopharmaceutical production. By leveraging host-specific translational mechanisms, this strategy can significantly improve the expression of recombinant proteins, including enzymes, therapeutic antibodies, and vaccines. Such improvements not only enhance production efficiency but also reduce costs and timelines, ultimately facilitating the development of more effective bioproducts. In conclusion, codon optimization should move beyond single-metric strategies to embrace integrated approaches that account for the interplay of multiple factors. This paradigm shift will enable researchers to design sequences with higher translational efficiency and expression stability, paving the way for advances in synthetic biology, industrial biotechnology, and precision biopharmaceutical manufacturing.

## Supplemental Materials

Supplementary data for this paper are available on-line only at http://jmb.or.kr.



## Figures and Tables

**Table 1:** Design criteria, parameters and constraints supported by codon optimization tools.

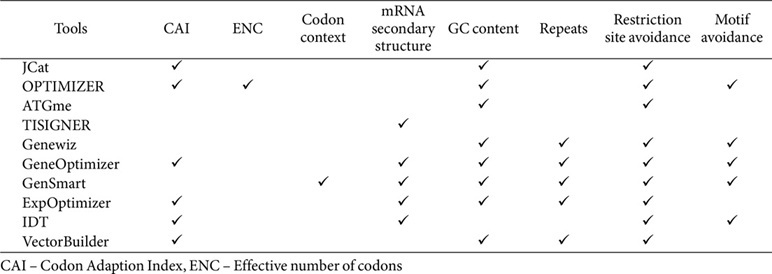

**Table 2:** Information of target proteins.

Protein	Sequence length (amino acids)	NCBI info	Host organism	Application
Insulin	110	NM_001185098.2	*E. coli*	Hormones used to treat diabetes
α-Amylase	622	XM_013469492.1	*S. cerevisiae*	Enzymes used to degrade starch in food, bioethanol, etc.
Humira, heavy chain	445	LQ506328.1	CHO	Monoclonal antibody used to treat autoimmune conditions.
Humira, light chain	215	LQ506329.1	CHO	
