# Supplementary material for: Comparative Analysis of Codon Optimization Tools: Advancing toward a Multi-Criteria Framework for Synthetic Gene Design
Source: J Microbiol Biotechnol. 2025 Apr 10;35:e2411066. doi: 10.4014/jmb.2411.11066 (PMC12010093; doi:10.4014/jmb.2411.11066)
Supplement: Supplementary file 1 [file jmb-35-e2411066-supple.pdf]

**Table S1: Codon optimization tools:** The data was collected until June 2024**.** * A – Academia, C – Commercial (company name)

| Type | Tools | Ownership | Year | Availability |
|---|---|---|---|---|
| **Stand-alone** | GMAP | A | 1994 | Upon request |
| | Codon Optimizer | A | 2003 | N/A |
| | GeMS | A | 2005 | N/A |
| | GASCO | A | 2008 | N/A |
| | Visual gene developer | A | 2011 | http://www.visualgenedeveloper.net/Intro_VGD.html |
| | Eugene | A | 2012 | N/A |
| | COStar | A | 2014 | N/A |
| | TaiCO | A | 2016 | https://2016.igem.org/Team:DTU-Denmark/Software |
| | CodonWizard | A | 2019 | http://schwalbe.org.chemie.uni-frankfurt.de/node/3324 |
| | Codon pair Optimization (CPO) | A | 2021 | N/A |
| **Toolbox** | D-tailor | A | 2014 | https://sourceforge.net/projects/dtailor/ |
| | MOOLTi | A | - | http://2018.igem.org/Team:HebrewU/Software |
| **Web-based** | DNAWorks | A | 2002 | https://hpcwebapps.cit.nih.gov/dnaworks/ |
| | Gene2Oligo | A | 2004 | N/A |
| | Upgene | A | 2004 | N/A |
| | JCat | A | 2005 | http://www.jcat.de/ |
| | GeneDesign | A | 2006 | N/A |
| | Synthetic gene designer | A | 2006 | N/A |
| | DyNAVacs | A | 2006 | N/A |
| | OPTIMIZER | A | 2007 | http://genomes.urv.es/OPTIMIZER/ |
| | CAIcal | A | 2008 | http://genomes.urv.es/CAIcal/ |
| | COOL | A | 2014 | N/A |
| | GeneGenie | A | 2014 | N/A |
| | ATGme | A | 2015 | http://atgme.org/ |
| | TISIGNER | A | 2021 | https://tisigner.com/ |
| | Wild Worm Codon Adapter | A | 2021 | https://hallemlab.shinyapps.io/Wild_Worm_Codon_Adapter/ |
| | Eurofins | C (Blue Heron Biotech LLC) | 1999 | https://www.blueheronbio.com/codon-optimization/ |
| | Genewiz | C (Genewiz) | 2010 | https://www.genewiz.com/Public/Services/Gene-Synthesis/Codon-Optimization/ |
| | GeneOptimizer | C (Thermofisher, merged with GeneArt) | 2010 | https://www.thermofisher.com/us/en/home/life-science/cloning/gene-synthesis.html |
| | GenSmart | C (GenScript) | - | https://www.genscript.com/gensmart-free-gene-codon-optimization.html |
| | ExpOptimizer | C (NovoPro) | - | https://www.novoprolabs.com/tools/codon-optimization |
| | IDT | C (IDT) | - | https://sg.idtdna.com/pages/tools/codon-optimization-tool |
| | VectorBuilder | C (VectorBuilder) | - | https://en.vectorbuilder.com/tool/codon-optimization.html |
| | EnCor | C (Encor Biotechnology Inc.) | - | http://www.encorbio.com/protocols/Codon.htm |

**Table S2.** Calculated values of the Wild type sequence of Human insulin before and after optimization provided by tools vs our calculated value. **HE**, highly expressed

| 1.Human insulin (INS), NM_001185098.2 | | | | | | | | | | | |
|---|---|---|---|---|---|---|---|---|---|---|---|
| **Tools** | **Tool provided** | | | | **Our calculated value using the selected formulas** | | | | | | |
| | **Before optimization** | | **After optimization** | | | | | | | | |
| | **CAI** | **GC content** | **CAI** | **GC content** | **CAI (HE)** | **CAI (Genome)** | **CBP score** | **GC content** | **mRNA free energy (Kcal/mol)** | | |
| | | | | | | | | | **RNAFold** | **UNAfold** | **RNAstructure** |
| JCat | 0.296 | 64.56 | 1 | 56.76 | 0.989 | 0.886 | 0 | 56.76 | -122.3 | -124.4 | -124.6 |
| OPTIMIZER | 0.436 | 64.60 | 1 | 60.10 | 0.974 | 0.914 | 0 | 60.06 | -120.3 | -127.3 | -126.2 |
| ATGme | - | 64.56 | - | 64.26 | 0.940 | 0.985 | 0 | 64.26 | -156.5 | -160.8 | -159.9 |
| TISIGNER | - | - | - | - | 0.576 | 0.789 | 0.555 | 62.76 | -142.2 | -146.1 | -142.8 |
| Genewiz | - | - | - | - | 0.791 | 0.927 | 0.346 | 60.96 | -142.2 | -145.3 | -143.8 |
| GeneOptimizer | - | - | - | - | 0.914 | 0.903 | 0.191 | 51.95 | -111.7 | -117.9 | -113.5 |
| GenSmart | - | 64.56 | - | 56.46 | 0.700 | 0.837 | 0.628 | 56.46 | -118.5 | -127.5 | -122.5 |
| ExpOptimizer | 0.480 | 64.56 | 0.870 | 58.86 | 0.914 | 0.882 | 0.286 | 58.86 | -120.9 | -127.3 | -122.2 |
| IDT | - | - | - | - | 0.522 | 0.781 | 0.615 | 51.05 | -104 | -108.1 | -105.4 |
| VectorBuilder | - | - | 0.920 | 59.76 | 0.938 | 0.943 | 0.257 | 59.76 | -137.2 | -144.4 | -138.3 |
| Wild type | | | | | 0.599 | 0.796 | 0.506 | 64.56 | -147.2 | -148.3 | -150.8 |

**Table S3.** Calculated values of the Wild type sequence of α-amylase before and after optimization provided by tools vs our calculated value. **HE**, highly expressed

<table>
<tr><th colspan="14">2. α-amylase, XM_013469492.1</th></tr>
<tr><th rowspan="4">Tools</th><th colspan="4">Tool provided</th><th colspan="9" rowspan="2">Our calculated value using the selected formulas</th></tr>
<tr><th colspan="2">Before optimization</th><th colspan="2">After optimization</th></tr>
<tr><th rowspan="2">CAI</th><th rowspan="2">GC content</th><th rowspan="2">CAI</th><th rowspan="2">GC content</th><th rowspan="2">CAI (HE)</th><th rowspan="2">CAI (Genome)</th><th rowspan="2">CBP score</th><th rowspan="2">GC content</th><th colspan="3">mRNA free energy (Kcal/mol)</th></tr>
<tr><th>RNAFold</th><th>UNAfold</th><th>RNAstructure</th></tr>
<tr><td>JCat</td><td>0.059</td><td>52.38</td><td>0.936</td><td>42.75</td><td>0.951</td><td>0.828</td><td>0.010</td><td>42.75</td><td>-466.1</td><td>-489.2</td><td>-470.8</td></tr>
<tr><td>OPTIMIZER</td><td>0.607</td><td>52.40</td><td>1</td><td>32.50</td><td>0.956</td><td>0.974</td><td>0</td><td>32.48</td><td>-397.7</td><td>-414</td><td>-402.5</td></tr>
<tr><td>ATGme</td><td>-</td><td>52.38</td><td>-</td><td>32.42</td><td>0.958</td><td>0.974</td><td>0</td><td>32.42</td><td>-397.7</td><td>-413.9</td><td>-402.5</td></tr>
<tr><td>TISIGNER</td><td>-</td><td>-</td><td>-</td><td>-</td><td>0.464</td><td>0.693</td><td>0.547</td><td>52.27</td><td>-627.9</td><td>-653</td><td>-633</td></tr>
<tr><td>Genewiz</td><td>-</td><td>-</td><td>-</td><td>-</td><td>0.851</td><td>0.917</td><td>0.332</td><td>35.79</td><td>-431.2</td><td>-453.9</td><td>-437.4</td></tr>
<tr><td>GeneOptimizer</td><td>-</td><td>-</td><td>-</td><td>-</td><td>0.891</td><td>0.866</td><td>0.273</td><td>40.56</td><td>-452.1</td><td>-476.2</td><td>-457.1</td></tr>
<tr><td>GenSmart</td><td>-</td><td>52.38</td><td>-</td><td>45.26</td><td>0.838</td><td>0.799</td><td>0.284</td><td>45.26</td><td>-469.6</td><td>-506.3</td><td>-477.1</td></tr>
<tr><td>ExpOptimizer</td><td>0.650</td><td>52.38</td><td>0.800</td><td>41.14</td><td>0.627</td><td>0.820</td><td>0.483</td><td>41.14</td><td>-489</td><td>-511.3</td><td>-493.2</td></tr>
<tr><td>IDT</td><td>-</td><td>-</td><td>-</td><td>-</td><td>0.524</td><td>0.756</td><td>0.530</td><td>46.23</td><td>-556.1</td><td>-583.3</td><td>-560.5</td></tr>
<tr><td>VectorBuilder</td><td>0.610</td><td>52.38</td><td>0.870</td><td>38.20</td><td>0.775</td><td>0.873</td><td>0.402</td><td>38.20</td><td>-447.6</td><td>-472.3</td><td>-452.6</td></tr>
<tr><td>Wild type</td><td></td><td></td><td></td><td></td><td>0.460</td><td>0.796</td><td>0.548</td><td>52.38</td><td>-632.9</td><td>-658.6</td><td>-636.5</td></tr>
</table>

**Table S4.** Calculated values of the Wild type sequence of Adalimumab (Humira) Heavy Chain before and after optimization provided by tools vs our calculated value. **HE**, highly expressed

| 3.1 Adalimumab (Humira) Heavy Chain, LQ506328.1 | | | | | | | | | | | |
|---|---|---|---|---|---|---|---|---|---|---|---|
| **Tools** | **Tool provided** | | | | **Our calculated value using the selected formulas** | | | | | | |
| | **Before optimization** | | **After optimization** | | | | | | | | |
| | **CAI** | **GC content** | **CAI** | **GC content** | **CAI (HE)** | **CAI (Genome)** | **CBP score** | **GC content** | **mRNA free energy (Kcal/mol)** | | |
| | | | | | | | | | **RNAFold** | **UNAfold** | **RNAstructure** |
| JCat | - | - | - | - | - | - | - | - | - | - | - |
| OPTIMIZER | 0.725 | 44.91 | 1 | 62 | 0.997 | 0.760 | 0.031 | 61.90 | -533.9 | -555.2 | -540.7 |
| ATGme | - | 44.91 | - | 66.08 | 0.978 | 0.739 | 0 | 66.08 | -550.2 | -572 | -557.5 |
| TISIGNER | - | - | - | - | - | - | - | - | - | - | - |
| Genewiz | - | - | - | - | 0.948 | 0.749 | 0.261 | 60.59 | -493 | -513.7 | -497.2 |
| GeneOptimizer | - | - | - | - | 0.945 | 0.782 | 0.336 | 57.73 | -475.3 | -496.8 | -479.5 |
| GenSmart | - | 44.91 | - | 57.58 | 0.924 | 0.769 | 0.332 | 57.58 | -472.7 | -487.8 | -474.9 |
| ExpOptimizer | 0.700 | 44.91 | 0.830 | 52.23 | 0.829 | 0.771 | 0.533 | 52.23 | -452.3 | -465 | -458 |
| IDT | - | - | - | - | 0.763 | 0.771 | 0.545 | 48.94 | -379.7 | -394.9 | -380 |
| VectorBuilder | 0.690 | 44.91 | 0.930 | 57.22 | 0.934 | 0.776 | 0.396 | 57.22 | -501.1 | -516.4 | -506.6 |
| Wild type | | | | | 0.711 | 0.785 | 0.601 | 44.91 | -358.6 | -377.2 | -364.5 |

**Table S5.** Calculated values of the Wild type sequence of Adalimumab (Humira) Light Chain before and after optimization provided by tools vs our calculated value. **HE**, highly expressed

| 3.2 Adalimumab (Humira) Light Chain, LQ506329.1 | | | | | | | | | | | |
|---|---|---|---|---|---|---|---|---|---|---|---|
| **Tools** | **Tool provided** | | | | **Our calculated value using the selected formulas** | | | | | | |
| | **Before optimization** | | **After optimization** | | | | | | | | |
| | **CAI** | **GC content** | **CAI** | **GC content** | **CAI (HE)** | **CAI (Genome)** | **CBP score** | **GC content** | **mRNA free energy (Kcal/mol)** | | |
| | | | | | | | | | **RNAFold** | **UNAfold** | **RNAstructure** |
| JCat | - | - | - | - | - | - | - | - | - | - | - |
| OPTIMIZER | 0.745 | 43.60 | 1 | 61.60 | 1 | 0.742 | 0 | 61.55 | -255.0 | -232.8 | -230.5 |
| ATGme | - | 44.57 | - | 65.43 | 0.978 | 0.725 | 0 | 65.43 | -238.7 | -250 | -242.5 |
| TISIGNER | - | - | - | - | - | - | - | - | - | - | - |
| Genewiz | - | - | - | - | 0.957 | 0.741 | 0.215 | 59.53 | -217.4 | -228.1 | -221.4 |
| GeneOptimizer | - | - | - | - | 0.968 | 0.746 | 0.304 | 58.91 | -221.3 | -229.8 | -227.6 |
| GenSmart | - | 44.57 | - | 57.52 | 0.937 | 0.746 | 0.379 | 57.52 | -205.6 | -215.5 | -208.2 |
| ExpOptimizer | 0.700 | 44.57 | 0.840 | 52.56 | 0.827 | 0.748 | 0.618 | 52.56 | -200.0 | -210.4 | -199.5 |
| IDT | - | - | - | - | 0.784 | 0.740 | 0.627 | 49.77 | -174.6 | -184.7 | -181.9 |
| VectorBuilder | 0.700 | 44.57 | 0.940 | 58.29 | 0.941 | 0.749 | 0.461 | 58.29 | -218.7 | -226.6 | -219.9 |
| Wild type | | | | | 0.718 | 0.809 | 0.667 | 44.57 | -150.1 | -166.3 | -151.5 |

Sequence information of Wild type (**Red**) and optimized sequences (**Black**) from tools according to their order in the table.

## 1. Human insulin

ATGGCTCTGTGGATGCGTCTGCTGCCGCTGCTGGCTCTGCTGGCTCTGTGGGGTCCGGACCC
GGCTGCTGCTTTCGTTAACCAGCACCTGTGCGGTTCTCACCTGGTTGAAGCTCTGTACCTGG
TTTGCGGTGAACGTGGTTTCTTCTACACCCCGAAAACCCGTCGTGAAGCTGAAGACCTGCA
GGTTGGTCAGGTTGAACTGGGTGGTGGTCCGGGTGCTGGTTCTCTGCAGCCGCTGGCTCTG
GAAGGTTCTCTGCAGAAACGTGGTATCGTTGAACAGTGCTGCACCTCTATCTGCTCTCTGTA
CCAGCTGGAAAACTACTGCAACTAA

ATGGCGCTGTGGATGCGTCTGCTGCCGCTGCTGGCGCTGCTGGCGCTGTGGGGTCCGGAC
CCGGCGGCGGCGTTCGTTAACCAGCACCTGTGCGGTTCTCACCTGGTTGAAGCGCTGTACC
TGGTTTGCGGTGAACGTGGTTTCTTCTACACCCCGAAAACCCGTCGTGAAGCGGAAGACC
TGCAGGTTGGTCAGGTTGAACTGGGTGGTGGTCCGGGTGCGGGTTCTCTGCAGCCGCTGG
CGCTGGAAGGTTCTCTGCAGAAACGTGGTATCGTTGAACAGTGCTGCACCTCTATCTGCTC
TCTGTACCAGCTGGAAAACTACTGCAACTAG

ATGGCGCTGTGGATGCGCCTGCTGCCGCTGCTGGCGCTGCTGGCGCTGTGGGGCCCGGATC
CGGCGGCGGCGTTTGTGAACCAGCATCTGTGCGGCAGCCATCTGGTGGAAGCGCTGTATCT
GGTGTGCGGCGAACGCGGCTTTTTTTATACCCCGAAAACCCGCCGCGAAGCGGAAGATCTG
CAGGTGGGCCAGGTGGAACTGGGCGGCGGCCCGGGCGCGGGCAGCCTGCAGCCGCTGGC
GCTGGAAGGCAGCCTGCAGAAACGCGGCATTGTGGAACAGTGCTGCACCAGCATTTGCAG
CCTGTATCAGCTGGAAAACTATTGCAACTAA

ATGGCGCTCTGGATGCGATTATTACCTCTGCTGGCGCTGCTGGCCCTCTGGGGACCTGACCC
AGCCGCAGCCTTTGTGAACCAACACCTGTGCGGCTCACACCTGGTGGAAGCTCTCTACCTA
GTGTGCGGGGAACGAGGCTTCTTCTACACACCCAAGACCCGCCGGGAGGCAGAGGACCTG
CAGGTGGGGCAGGTGGAGCTGGGCGGGGGCCCTGGTGCAGGCAGCCTGCAGCCCTTGGCC
CTGGAGGGGTCCCTGCAGAAGCGTGGCATTGTGGAACAATGCTGTACCAGCATCTGCTCCC
TCTACCAGCTGGAGAACTACTGCAACTAG

ATGGCGCTGTGGATGCGGCTGCTGCCCCTGCTTGCTCTGCTGGCCCTTTGGGGGCCTGATCC
GGCGGCGGCGTTTGTGAATCAGCATCTGTGCGGCAGCCATCTGGTGGAAGCGCTGTATCTG
GTGTGCGGCGAACGCGGCTTTTTTTATACCCCGAAAACCCGCCGCGAAGCGGAAGATCTGC
AAGTGGGCCAAGTGGAATTGGGAGGCGGTCCGGGTGCCGGCAGTCTGCAGCCGCTGGCGC
TGGAAGGCAGCCTGCAGAAGCGCGGCATTGTGGAACAGTGCTGCACGAGCATTTGCAGCC
TGTATCAGCTGGAAAACTATTGCAACTAA

ATGGCACTGTGGATGCGTCTGCTGCCGCTGCTGGCACTGCTGGCCCTGTGGGGTCCTGATCC
GGCAGCAGCATTTGTTAATCAGCATCTGTGTGGTAGCCATCTGGTTGAAGCACTGTATCTGG
TTTGTGGTGAACGTGGTTTCTTTTATACCCCGAAAACACGTCGTGAAGCAGAAGATCTGCA
GGTTGGTCAGGTTGAATTAGGTGGTGGTCCTGGTGCAGGTAGCCTGCAGCCGCTGGCGCTG
GAAGGTAGTCTGCAGAAACGTGGTATTGTTGAACAGTGTTGTACCAGCATTTGCAGCCTGT
ATCAGCTGGAAAACTATTGCAACTAA

ATGGCTCTATGGATGAGGTTACTTCCCTTGTTGGCGTTGTTGGCGCTGTGGGGTCCAGATCC
GGCAGCTGCCTTTGTTAATCAGCACCTTTGCGGCTCCCATCTGGTCGAGGCTCTCTACCTGG
TGTGCGGTGAACGTGGCTTCTTCTATACCCCGAAAACGCGTCGTGAAGCGGAAGACCTGCA
AGTGGGCCAGGTTGAGCTGGGTGGCGGTCCGGGTGCAGGCTCTCTGCAACCGCTGGCGCT
GGAAGGTAGCCTGCAAAAGCGCGGTATTGTTGAGCAGTGTTGTACCAGCATCTGCAGCTTA
TACCAGCTGGAGAACTATTGCAACTAA

ATGGCTCTGTGGATGCGTCTGCTGCCGCTGCTGGCACTGCTGGCCCTGTGGGGTCCGGACC
CGGCCGCAGCGTTCGTTAACCAGCACCTGTGTGGCTCTCACCTGGTAGAAGCCCTGTACCT
GGTATGCGGTGAACGTGGTTTCTTCTACACCCCTAAAACTCGTCGCGAAGCAGAAGACCTG
CAGGTTGGCCAGGTTGAACTGGGTGGTGGTCCAGGTGCTGGTAGCCTGCAGCCGCTGGCA
CTGGAGGGTTCTCTGCAGAAACGCGGTATCGTTGAGCAGTGCTGCACCTCTATTTGCTCCCT
GTACCAGCTGGAAAACTACTGCAACTAA

ATGGCCCTGTGGATGCGTCTGCTGCCTTTATTAGCATTGTTAGCTCTTTGGGGGCCCGATCCC
GCTGCAGCTTTTGTAAATCAGCATTTGTGTGGGTCACATCTGGTTGAAGCGTTATACCTTGTA
TGCGGGGAACGCGGGTTTTTCTACACTCCCAAGACCCGCCGTGAGGCCGAGGATCTTCAAG
TAGGACAGGTTGAATTGGGCGGAGGGCCAGGTGCGGGTTCCTTGCAACCTCTGGCGTTAGA
GGGAAGCCTTCAGAAACGCGGAATCGTAGAACAATGTTGCACCTCAATCTGTTCTTTATATC
AGCTTGAGAATTACTGTAACTAG

ATGGCCCTGTGGATGCGTCTGCTGCCGCTGCTGGCGCTGCTGGCGCTGTGGGGTCCGGATC
CGGCCGCGGCGTTTGTGAACCAGCATCTGTGCGGTAGCCATCTGGTTGAAGCGCTGTATCTG
GTGTGCGGTGAACGTGGCTTTTTCTACACCCCGAAAACCCGCCGCGAAGCAGAAGATTTAC
AGGTGGGCCAGGTTGAACTGGGTGGCGGCCCGGGTGCAGGCAGTCTGCAGCCGCTGGCGC
TGGAAGGTAGCCTGCAGAAACGTGGCATTGTTGAACAGTGTTGCACCAGCATTTGCAGCCT
GTACCAGCTGGAAAACTATTGCAACTAA

ATGGCCCTGTGGATGCGCCTCCTGCCCCTGCTGGCGCTGCTGGCCCTCTGGGGACCTGACC
CAGCCGCAGCCTTTGTGAACCAACACCTGTGCGGCTCACACCTGGTGGAAGCTCTCTACCT
AGTGTGCGGGGAACGAGGCTTCTTCTACACACCCAAGACCCGCCGGGAGGCAGAGGACCT
GCAGGTGGGGCAGGTGGAGCTGGGCGGGGGCCCTGGTGCAGGCAGCCTGCAGCCCTTGGC

CCTGGAGGGGTCCCTGCAGAAGCGTGGCATTGTGGAACAATGCTGTACCAGCATCTGCTCC
CTCTACCAGCTGGAGAACTACTGCAACTAG

2. **α-amylase**

ATGACTCCATTCGTTTTGACTGCTGTTTTGTTCTTGTTGGGTAACGCTGTTTTGGCTTTGACT
CCAGCTGAATGGAGAAAGCAATCTATCTACTTCTTGTTGACTGACAGATTCGGTAGAGCTGA
CAACTCTACTACTGCTGCTTGTGACGTTACTGAAAGAATCTACTGTGGTGGTTCTTGGCAAG
GTATCATCAACCACTTGGACTACATCCAAGGTATGGGTTTCACTGCTATCTGGATCTCTCCAG
TTACTGAACAATTGCCACAAAACACTGGTGAAGGTGAAGCGTACCACGGTTACTGGCAACA
AGAAATCTACACTGTTAACTCTAACTTCGGTACTTCTGACGACTTGTTGGCTTTGTCTAAGG
CTTTGCACGACAGAGGTATGTACTTGATGGTTGACGTTGTTGCTAACCACATGGGTTACGAC
GGTGACGGTGACTCTGTTGACTACTCTGTTTTCAACCCATTCAACTCTTCTTCTTACTTCCAC
CCATACTGTTTGATCACTGACTACTCTAACCAAACTGACGTTGAAGACTGTTGGTTGGGTGA
CACTACTGTTTCTTTGCCAGACTTGAACACTACTGAAACTGTTGTTAGAACTATCTGGTACG
ACTGGGTTGCTGACTTGGTTTCTAACTACTCTATCGACGGTTTGAGAATCGACACTGTTAAG
CACGTTGAAAAGTCTTTCTGGCCAGGTTACAACTCTGCTGCTGGTGTTTACTGTGTTGGTGA
AGTTTTGGACGGTGACCCATCTTACACTTGTCCATACCAAGACTACTTGGACGGTGTTTTGA
ACTACCCAATCTACTACCAATTGTTGTACGCTTTCGAATCTTCTTCTGGTTCTATCTCTAACTT
GTACAACATGATCAACTCTGTTGCTTCTGAATGTTCTGACCCAACTTTGTTGGGTAACTTCAT
CGAAAACCACGACAACCCAAGATTCGCTTCTTACACTTCTGACTACTCTTTGGCTAAGAAC
GTTATCGCTTTCATCTTCTTCTCTGACGGTATCCCAATCGTTTACGCTGGTCAAGAACAACAC
TACAACGGTGGTAACGACCCATACAACAGAGAAGCTACTTGGTTGTCTGGTTACTCTACTAC
TGCTGAATTGTACACTTTCATCGCTACTACTAACGCTATCAGATCTTTGGCTATCTCTGTTGA
CTCTGAATACTTGACTTACAAGAACGACCCATTCTACTACGACTCTAACACTTTGGCTATGA
GAAAGGGTTCTGACGGTTTGCAAGTTATCACTGTTTTGTCTAACTTGGGTGCTGACGGTTCT
TCTTACACTTTGACTTTGTCTGGTTCTGGTTACTCTTCTGGTACTGAATTGGTTGAAGCGTAC
ACTTGTACTACTGTTACTGTTGACTCTAACGGTGACATCCCAGTTCCAATGGAATCTGGTTTG
CCAAGAGTTTTCTTGCCAGCTTCTTCTTTCTCTGGTTCTTCTTTGTGTTCTTCTTCTCCATCTC
CAACTACTACTACTTCTACTTCTACTTCTACTACTTCTACTGCTTGTACTACTGCTACTGCTGT
TGCTGTTTTGTTCGAAGAATTGGTTACTACTACTTACGGTGAAAACGTTTACTTGTCTGGTTC
TATCTCTCAATTGGGTGACTGGAACACTGACGACGCTGTTGCTTTGTCTGCTGCTAACTACA
CTTCTTCTAACCCATTGTGGTACGTTACTGTTACTTTGCCAGTTGGTACTTCTTTCGAATACA
AGTTCATCAAGAAGGAAGAAAACGGTGACGTTGAATGGGAATCTGACCCAAACAGATCTT
ACACTGTTCCAACTGCTTGTACTGGTGCTACTGAAACTATCGTTGACACTTGGAGATAA

ATGACTCCATTTGTTTTGACTGCTGTTTTGTTTTTGTTGGGTAATGCTGTTTTGGCTTTGACT
CCAGCTGAATGGAGAAAACAATCTATTTATTTTTTGTTGACTGATAGATTTGGTAGAGCTGAT
AATTCTACTACTGCTGCTTGTGATGTTACTGAAAGAATTTATTGTGGTGGTTCTTGGCAAGGT

ATTATTAATCATTTGGATTATATTCAAGGTATGGGTTTTACTGCTATTTGGATTTCTCCAGTTAC
TGAACAATTGCCACAAAATACTGGTGAAGGTGAAGCTTATCATGGTTATTGGCAACAAGAA
ATTTATACTGTTAATTCTAATTTTGGTACTTCTGATGATTTGTTGGCTTTGTCTAAAGCTTTGC
ATGATAGAGGTATGTATTTGATGGTTGATGTTGTTGCTAATCATATGGGTTATGATGGTGATGG
TGATTCTGTTGATTATTCTGTTTTTAATCCATTTAATTCTTCTTCTTATTTTCATCCATATTGTTT
GATTACTGATTATTCTAATCAAACTGATGTTGAAGATTGTTGGTTGGGTGATACTACTGTTTC
TTTGCCAGATTTGAATACTACTGAAACTGTTGTTAGAACTATTTGGTATGATTGGGTTGCTGA
TTTGGTTTCTAATTATTCTATTGATGGTTTGAGAATTGATACTGTTAAACATGTTGAAAAATCT
TTTTGGCCAGGTTATAATTCTGCTGCTGGTGTTTATTGTGTTGGTGAAGTTTTGGATGGTGAT
CCATCTTATACTTGTCCATATCAAGATTATTTGGATGGTGTTTTGAATTATCCAATTTATTATCA
ATTGTTGTATGCTTTTGAATCTTCTTCTGGTTCTATTTCTAATTTGTATAATATGATTAATTCTGT
TGCTTCTGAATGTTCTGATCCAACTTTGTTGGGTAATTTTATTGAAAATCATGATAATCCAAG
ATTTGCTTCTTATACTTCTGATTATTCTTTGGCTAAAAATGTTATTGCTTTTATTTTTTTTCTG
ATGGTATTCCAATTGTTTATGCTGGTCAAGAACAACATTATAATGGTGGTAATGATCCATATAA
TAGAGAAGCTACTTGGTTGTCTGGTTATTCTACTACTGCTGAATTGTATACTTTTATTGCTACT
ACTAATGCTATTAGATCTTTGGCTATTTCTGTTGATTCTGAATATTTGACTTATAAAAATGATC
CATTTTATTATGATTCTAATACTTTGGCTATGAGAAAAGGTTCTGATGGTTTGCAAGTTATTAC
TGTTTTGTCTAATTTGGGTGCTGATGGTTCTTCTTATACTTTGACTTTGTCTGGTTCTGGTTAT
TCTTCTGGTACTGAATTGGTTGAAGCTTATACTTGTACTACTGTTACTGTTGATTCTAATGGTG
ATATTCCAGTTCCAATGGAATCTGGTTTGCCAAGAGTTTTTTTGCCAGCTTCTTCTTTTTCTG
GTTCTTCTTTGTGTTCTTCTTCTCCATCTCCAACTACTACTACTTCTACTTCTACTTCTACTAC
TTCTACTGCTTGTACTACTGCTACTGCTGTTGCTGTTTTGTTTGAAGAATTGGTTACTACTAC
TTATGGTGAAAATGTTTATTTGTCTGGTTCTATTTCTCAATTGGGTGATTGGAATACTGATGAT
GCTGTTGCTTTGTCTGCTGCTAATTATACTTCTTCTAATCCATTGTGGTATGTTACTGTTACTTT
GCCAGTTGGTACTTCTTTTGAATATAAATTTATTAAAAAAGAAGAAAATGGTGATGTTGAAT
GGGAATCTGATCCAAATAGATCTTATACTGTTCCAACTGCTTGTACTGGTGCTACTGAAACTA
TTGTTGATACTTGGAGATAG

ATGACTCCATTTGTTTTGACTGCTGTTTTGTTTTTGTTGGGTAATGCTGTTTTGGCTTTGACT
CCAGCTGAATGGAGAAAACAATCTATTTATTTTTTGTTGACTGATAGATTTGGTAGAGCTGAT
AATTCTACTACTGCTGCTTGTGATGTTACTGAAAGAATTTATTGTGGTGGTTCTTGGCAAGGT
ATTATTAATCATTTGGATTATATTCAAGGTATGGGTTTTACTGCTATTTGGATTTCTCCAGTTAC
TGAACAATTGCCACAAAATACTGGTGAAGGTGAAGCTTATCATGGTTATTGGCAACAAGAA
ATTTATACTGTTAATTCTAATTTTGGTACTTCTGATGATTTGTTGGCTTTGTCTAAAGCTTTGC
ATGATAGAGGTATGTATTTGATGGTTGATGTTGTTGCTAATCATATGGGTTATGATGGTGATGG
TGATTCTGTTGATTATTCTGTTTTTAATCCATTTAATTCTTCTTCTTATTTTCATCCATATTGTTT
GATTACTGATTATTCTAATCAAACTGATGTTGAAGATTGTTGGTTGGGTGATACTACTGTTTC
TTTGCCAGATTTGAATACTACTGAAACTGTTGTTAGAACTATTTGGTATGATTGGGTTGCTGA
TTTGGTTTCTAATTATTCTATTGATGGTTTGAGAATTGATACTGTTAAACATGTTGAAAAATCT
TTTTGGCCAGGTTATAATTCTGCTGCTGGTGTTTATTGTGTTGGTGAAGTTTTGGATGGTGAT

CCATCTTATACTTGTCCATATCAAGATTATTTGGATGGTGTTTTGAATTATCCAATTTATTATCA
ATTGTTGTATGCTTTTGAATCTTCTTCTGGTTCTATTTCTAATTTGTATAATATGATTAATTCTGT
TGCTTCTGAATGTTCTGATCCAACTTTGTTGGGTAATTTTATTGAAAATCATGATAATCCAAG
ATTTGCTTCTTATACTTCTGATTATTCTTTGGCTAAAAATGTTATTGCTTTTATTTTTTTTTCTG
ATGGTATTCCAATTGTTTATGCTGGTCAAGAACAACATTATAATGGTGGTAATGATCCATATAA
TAGAGAAGCTACTTGGTTGTCTGGTTATTCTACTACTGCTGAATTGTATACTTTTATTGCTACT
ACTAATGCTATTAGATCTTTGGCTATTTCTGTTGATTCTGAATATTTGACTTATAAAAATGATC
CATTTTATTATGATTCTAATACTTTGGCTATGAGAAAAGGTTCTGATGGTTTGCAAGTTATTAC
TGTTTTGTCTAATTTGGGTGCTGATGGTTCTTCTTATACTTTGACTTTGTCTGGTTCTGGTTAT
TCTTCTGGTACTGAATTGGTTGAAGCTTATACTTGTACTACTGTTACTGTTGATTCTAATGGTG
ATATTCCAGTTCCAATGGAATCTGGTTTGCCAAGAGTTTTTTTGCCAGCTTCTTCTTTTTCTG
GTTCTTCTTTGTGTTCTTCTTCTCCATCTCCAACTACTACTACTTCTACTTCTACTTCTACTAC
TTCTACTGCTTGTACTACTGCTACTGCTGTTGCTGTTTTGTTTGAAGAATTGGTTACTACTAC
TTATGGTGAAAATGTTTATTTGTCTGGTTCTATTTCTCAATTGGGTGATTGGAATACTGATGAT
GCTGTTGCTTTGTCTGCTGCTAATTATACTTCTTCTAATCCATTGTGGTATGTTACTGTTACTTT
GCCAGTTGGTACTTCTTTTGAATATAAATTTATTAAAAAAGAAGAAAATGGTGATGTTGAAT
GGGAATCTGATCCAAATAGATCTTATACTGTTCCAACTGCTTGTACTGGTGCTACTGAAACTA
TTGTTGATACTTGGAGATAA

ATGACCCCCTTTGTCCTGACCGCCGTTTTGTTCTTGCTGGGGAATGCCGTGTTGGCCTTGAC
CCCGGCCGAATGGCGCAAACAATCTATCTACTTTCTCCTCACGGACCGCTTTGGCAGGGCA
GATAACTCGACCACTGCTGCCTGCGATGTCACTGAGAGGATCTACTGTGGCGGGAGTTGGC
AAGGAATCATCAACCATCTCGACTATATCCAAGGCATGGGGTTCACGGCCATCTGGATTTCA
CCGGTGACCGAGCAGCTGCCGCAAAATACGGGTGAGGGAGAAGCCTATCATGGGTATTGGC
AGCAGGAAATATACACGGTCAACTCCAACTTTGGGACATCAGACGATCTCTTAGCCCTGTCA
AAGGCGCTCCATGACCGTGGCATGTACCTCATGGTCGATGTGGTTGCGAATCACATGGGATA
CGATGGAGATGGCGACTCCGTTGATTACAGCGTCTTCAATCCATTTAATTCCTCGAGTTATTT
CCATCCCTATTGCCTGATTACAGACTACAGCAATCAGACCGATGTGGAAGACTGTTGGCTGG
GCGATACGACTGTCTCGTTGCCCGATCTCAACACCACGGAGACTGTTGTGAGGACTATATGG
TATGACTGGGTGGCGGATCTCGTCTCCAATTACTCTATTGATGGGCTTCGCATCGACACGGT
GAAACACGTAGAAAAGTCATTCTGGCCTGGTTACAACAGTGCTGCGGGTGTCTACTGTGTT
GGCGAGGTCCTCGATGGAGATCCGTCTTACACTTGTCCCTACCAGGATTATCTGGACGGTGT
ATTAAACTATCCAATATACTATCAACTACTGTATGCGTTTGAATCCTCTAGCGGCAGCATCAG
CAATCTTTACAACATGATCAACTCTGTCGCCTCTGAATGTTCCGATCCCACTCTGTTGGGCA
ACTTTATCGAGAACCATGACAACCCTAGATTTGCCTCCTATACAAGTGATTATTCTCTTGCTA
AAAATGTGATTGCTTTCATCTTCTTCTCTGACGGCATCCCTATCGTCTATGCCGGTCAGGAGC
AGCATTACAACGGGGGAAATGACCCCTACAACCGCGAGGCCACCTGGCTGTCAGGATACTC
GACGACGGCCGAACTGTACACGTTCATTGCGACCACCAACGCGATCCGTAGCTTGGCGATC
TCCGTCGACTCGGAGTATTTGACGTACAAGAATGACCCATTCTACTACGACAGCAATACCCT
CGCTATGCGCAAGGGTTCGGATGGCCTGCAGGTCATCACTGTTCTGTCCAATCTGGGCGCCG

ATGGTAGCTCGTACACGTTGACTCTGAGTGGCAGTGGCTATTCGTCAGGCACGGAGCTGGT
GGAAGCTTACACCTGCACAACGGTCACTGTTGACTCTAATGGCGATATTCCAGTTCCCATGG
AGTCCGGACTGCCGCGCGTTTTCCTACCAGCATCCTCATTCAGTGGTAGCAGTCTATGCAGT
TCTTCTCCTAGCCCTACTACTACAACATCGACATCGACATCGACAACGTCGACGGCCTGCAC
CACCGCCACCGCTGTGGCGGTCCTCTTCGAAGAGTTGGTGACAACGACCTACGGTGAAAAT
GTCTACCTCAGCGGATCGATCAGCCAACTCGGGGACTGGAACACGGACGACGCCGTGGCC
CTGTCCGCAGCTAATTACACTTCTTCGAATCCCCTGTGGTATGTGACAGTCACATTGCCGGTT
GGGACGTCCTTTGAGTACAAGTTCATCAAGAAGGAAGAGAACGGCGATGTCGAGTGGGAG
AGCGATCCCAATCGGTCGTATACTGTGCCGACGGCCTGCACGGGAGCGACGGAGACGATTG
TCGACACATGGAGATAG

ATGACTCCATTTGTTTTGACTGCCGTTTTGTTTCTCTTGGGAAATGCAGTCTTGGCTTTGACT
CCTGCTGAATGGAGAAAACAATCTATTTATTTTTTGTTGACTGATAGATTTGGTAGAGCTGAT
AATTCGACCACGGCTGCTTGCGATGTTACTGAAAGAATTTATTGTGGTGGTTCTTGGCAAGG
TATTATTAATCATTTGGATTATATTCAAGGTATGGGTTTTACTGCTATTTGGATTTCTCCTGTTA
CTGAACAATTGCCACAAAATACTGGTGAAGGTGAAGCTTATCATGGTTATTGGCAACAAGA
AATTTATACTGTTAATTCTAATTTTGGTACTTCTGATGATTTGTTGGCTTTGTCTAAAGCTTTG
CATGATAGAGGTATGTATTTGATGGTTGATGTTGTTGCTAATCATATGGGTTATGATGGTGATG
GTGACTCTGTTGATTACTCTGTGTTTAACCCATTTAATTCCTCATCTTATTTCCATCCCTATTGC
CTTATTACTGACTATTCTAATCAAACTGATGTTGAAGATTGTTGGTTGGGTGATACTACTGTT
TCTTTGCCTGATTTGAATACTACTGAAACTGTTGTTAGAACTATTTGGTATGATTGGGTTGCT
GATTTGGTTAGCAACTATTCTATTGACGGTTTGAGAATAGATACTGTTAAACATGTTGAAAAA
TCTTTTTGGCCTGGTTATAATTCTGCTGCTGGTGTTTATTGTGTTGGTGAAGTTTTGGATGGT
GATCCATCTTATACTTGTCCGTACCAAGATTATTTGGATGGTGTTTTGAATTATCCAATTTACT
ATCAATTGTTGTATGCATTTGAATCTAGCTCTGGTTCTATTTCTAACTTGTATAATATGATTAAT
TCTGTTGCTTCTGAATGTTCTGACCCAACATTGTTAGGGAATTTTATTGAAAATCATGACAAT
CCAAGATTCGCTTCTTACACTTCGGATTATTCGTTGGCTAAAAACGTGATCGCATTTATTTTC
TTTTCTGATGGTATTCCAATTGTTTATGCTGGTCAAGAACAACATTATAATGGTGGTAATGATC
CATATAATAGAGAAGCTACTTGGTTGAGTGGGTACTCTACTACTGCCGAATTATATACTTTTAT
TGCTACTACTAACGCCATCAGATCCTTGGCTATTTCCGTTGATTCTGAGTATTTGACTTATAAA
AATGACCCATTCTATTATGACTCTAATACTTTGGCTATGAGAAAAGGTTCTGATGGTTTGCAA
GTTATTACTGTTTTGTCTAATTTGGGTGCTGATGGTAGTTCATACACTTTGACCCTCTCGGGT
TCTGGTTACTCTTCTGGTACTGAATTGGTTGAAGCTTATACCTGTACTACTGTTACCGTTGAT
TCTAATGGTGATATTCCTGTTCCAATGGAATCTGGTTTGCCAAGAGTTTTTTTGCCTGCTTCT
TCTTTTTCTGGTTCTTCTTTGTGTTCTTCTTCTCCATCTCCAACTACGACAACTTCTACTTCTA
CAAGTACTACTTCTACCGCTTGTACTACTGCGACTGCTGTTGCTGTTCTGTTTGAAGAGTTG
GTTACTACTACTTATGGTGAAAATGTTTATTTGAGTGGTTCTATATCTCAATTGGGTGATTGGA
ATACTGATGATGCTGTTGCTTTGTCTGCTGCTAATTATACTTCTTCTAATCCATTGTGGTATGT
TACCGTGACTTTGCCTGTTGGTACTTCGTTCGAATATAAGTTTATCAAAAAAGAAGAAAATG

GTGATGTTGAATGGGAATCTGATCCAAATAGATCTTATACTGTTCCAACTGCTTGTACTGGTG
CAACTGAAACTATTGTTGATACTTGGAGATAA

ATGACCCCATTTGTTTTGACCGCTGTTTTGTTTTTGTTGGGTAATGCTGTTTTGGCTTTGACT
CCAGCTGAATGGCGTAAACAATCTATCTACTTCTTGTTGACCGACAGATTTGGTAGAGCTGA
TAATTCTACTACTGCTGCTTGTGATGTTACCGAAAGAATCTATTGTGGTGGTTCTTGGCAAGG
TATCATCAACCATTTGGATTACATTCAAGGTATGGGTTTCACCGCTATTTGGATTTCTCCAGTT
ACTGAACAATTGCCACAAAACACTGGTGAAGGTGAAGCTTATCATGGTTATTGGCAACAAG
AAATCTACACCGTCAATTCCAATTTCGGTACTTCCGATGATTTGTTGGCTTTGTCTAAGGCAT
TGCATGATAGAGGTATGTACTTGATGGTTGACGTTGTTGCTAATCACATGGGTTATGATGGTG
ATGGTGACTCTGTTGATTACTCTGTTTTTAACCCCTTCAACTCCTCCTCTTACTTTCATCCATA
CTGCTTGATTACCGACTACTCCAATCAAACTGATGTTGAAGATTGCTGGTTGGGTGATACAA
CTGTTTCTTTGCCAGATTTGAACACTACCGAAACTGTTGTTAGAACCATCTGGTATGATTGG
GTTGCTGATTTGGTTTCCAACTACTCTATTGACGGTTTGAGAATCGATACCGTTAAGCACGTT
GAAAAGTCTTTTTGGCCAGGTTATAATTCTGCTGCTGGTGTTTATTGTGTTGGTGAAGTTTTG
GATGGTGATCCATCTTATACTTGCCCATACCAAGATTATTTGGACGGTGTTTTGAACTACCCA
ATCTACTATCAGTTGTTGTACGCCTTCGAATCTTCCTCTGGTTCTATTTCTAACCTGTACAACA
TGATCAACTCCGTTGCTTCTGAATGTTCTGATCCAACTTTGTTGGGCAACTTCATCGAAAAT
CATGACAATCCAAGATTCGCCTCTTATACCTCTGATTACTCCTTGGCTAAGAACGTTATTGCC
TTCATCTTTTTCTCCGATGGTATCCCAATAGTTTACGCTGGTCAAGAACAACATTACAACGGT
GGTAATGATCCATACAACAGAGAAGCTACTTGGTTGTCTGGTTATTCTACAACCGCTGAACT
GTACACTTTCATTGCTACTACCAACGCCATTAGATCTTTGGCCATTTCAGTTGACTCTGAGTA
CTTGACTTACAAGAACGACCCATTTTACTACGACTCTAACACTTTGGCTATGAGAAAAGGTT
CCGATGGCTTGCAAGTTATTACCGTTTTGTCTAATTTGGGTGCCGATGGTTCTTCTTACACTT
TGACTTTATCTGGTTCCGGTTACTCTTCTGGTACTGAATTGGTTGAAGCTTACACTTGTACTA
CCGTTACCGTTGATTCTAACGGTGATATTCCAGTTCCAATGGAATCTGGTTTGCCAAGAGTTT
TCTTGCCAGCATCTTCTTTTTCTGGATCCTCTTTGTGTTCTTCTTCTCCATCTCCAACTACCAC
TACTTCTACATCTACTTCCACAACTTCTACTGCTTGTACAACTGCTACAGCTGTTGCTGTTTT
ATTCGAAGAATTGGTTACTACCACCTACGGTGAAAACGTTTATTTGTCCGGTTCCATCTCTCA
ATTAGGTGATTGGAATACTGATGATGCTGTTGCTTTATCTGCTGCTAACTACACTTCTTCTAAT
CCATTGTGGTACGTTACTGTCACTTTGCCAGTTGGTACTTCTTTTGAGTACAAGTTCATCAAG
AAAGAAGAAAACGGCGACGTTGAATGGGAATCTGATCCTAATAGATCTTACACTGTTCCAA
CAGCTTGTACTGGTGCTACTGAAACTATCGTTGATACTTGGAGATAA

ATGACTCCATTTGTTTTGACTGCCGTCTTGTTCTTGTTAGGTAACGCTGTCTTGGCTTTGACC
CCTGCTGAATGGAGAAAGCAATCTATCTACTTCTTGTTGACTGACAGATTCGGTAGAGCTGA
CAACTCCACCACAGCTGCTTGTGATGTCACCGAAAGAATCTACTGTGGTGGTTCTTGGCAA
GGTATCATCAACCATTTGGACTACATCCAAGGTATGGGTTTCACTGCTATCTGGATTTCTCCA
GTTACTGAACAATTGCCACAAAACACTGGTGAAGGTGAAGCTTACCACGGTTACTGGCAAC

AAGAAATTTACACCGTTAACTCTAACTTCGGTACCTCTGATGACTTGTTGGCCTTGTCCAAA
GCTTTGCACGACAGAGGTATGTACTTAATGGTTGACGTCGTCGCCAACCACATGGGTTACGA
CGGTGATGGTGATTCTGTAGACTACTCTGTCTTCAACCCATTTAATTCATCTTCTTACTTCCAC
CCATACTGTTTGATCACCGATTACTCCAACCAAACTGATGTTGAAGACTGTTGGTTGGGTGA
CACCACTGTTTCCTTGCCAGACTTAAATACCACCGAAACTGTTGTTAGAACCATCTGGTATG
ACTGGGTTGCTGATTTGGTCTCCAACTATTCCATTGATGGTCTACGTATTGACACCGTTAAGC
ACGTCGAGAAGTCTTTCTGGCCGGGTTACAACTCTGCTGCTGGAGTCTACTGTGTTGGTGA
AGTTTTAGATGGTGACCCATCATACACTTGTCCATACCAAGATTATTTGGACGGTGTTCTCAA
CTACCCAATCTACTATCAACTGTTGTACGCTTTCGAATCTAGCTCTGGTTCCATATCGAACTT
ATACAACATGATCAACTCCGTTGCTTCTGAATGTTCTGACCCAACTTTGTTGGGTAACTTCAT
TGAAAATCATGATAACCCAAGATTTGCTTCCTACACCTCTGACTACTCGTTAGCTAAGAACG
TCATTGCTTTCATCTTCTTCTCCGATGGTATCCCAATTGTCTACGCCGGTCAAGAACAACACT
ACAACGGTGGTAACGACCCTTACAACAGAGAAGCTACCTGGTTGAGCGGCTACTCTACTAC
TGCTGAATTGTACACTTTCATTGCCACCACCAACGCCATCCGTTCTTTGGCCATTTCCGTCGA
TTCCGAATACTTGACTTACAAGAACGATCCATTCTACTACGATTCTAACACTTTGGCTATGAG
AAAGGGTTCTGACGGTTTGCAAGTTATCACTGTTCTTAGCAACTTGGGTGCTGACGGTTCTT
CTTACACTTTAACTTTATCCGGTTCTGGTTACTCTTCCGGTACTGAATTGGTCGAAGCTTACA
CCTGTACTACCGTCACTGTGGACTCTAACGGCGACATTCCAGTTCCAATGGAATCCGGTTTG
CCAAGAGTTTTCTTACCAGCTTCTTCCTTCTCTGGTTCCTCTTTGTGTTCCTCTTCTCCATCC
CCAACCACCACTACTTCTACCTCCACTTCCACCACTTCCACTGCCTGCACCACTGCTACCGC
GGTTGCCGTTTTGTTTGAAGAATTGGTCACCACAACCTACGGTGAAAACGTTTACCTATCTG
GTTCTATTTCTCAATTGGGTGACTGGAACACCGATGATGCTGTCGCTTTGTCTGCCGCTAACT
ACACCTCCTCGAATCCATTGTGGTACGTTACTGTCACTTTGCCAGTTGGTACTTCCTTCGAAT
ATAAATTCATCAAGAAGGAAGAAAACGGTGACGTGGAATGGGAATCTGACCCAAACAGAT
CCTATACCGTCCCAACCGCTTGCACCGGTGCCACTGAAACCATTGTTGACACTTGGCGTTGA
ATGACTCCTTTTGTATTAACCGCTGTATTATTCCTTTTAGGCAATGCTGTCCTAGCCCTGACAC
CTGCTGAATGGAGAAAACAATCTATTTACTTTTTGCTAACCGATAGGTTTGGTCGTGCTGATA
ATTCAACCACAGCTGCCTGTGATGTAACGGAGAGAATCTATTGTGGTGGTTCTTGGCAGGGT
ATTATTAACCATTTGGATTACATACAGGGAATGGGTTTTACTGCAATATGGATTTCTCCTGTAA
CTGAACAATTACCCCAAAACACAGGTGAGGGTGAAGCTTACCACGGATATTGGCAACAAGA
AATTTATACGGTTAACTCTAATTTTGGGACCTCCGATGATCTATTGGCATTATCTAAGGCGTTA
CATGACCGTGGTATGTATTTAATGGTTGATGTAGTTGCGAACCATATGGGTTATGATGGAGAT
GGCGACTCTGTGGATTATTCAGTTTTTAATCCATTTAATAGTTCAAGCTACTTCCATCCGTATT
GTTTGATCACTGACTATAGTAATCAGACCGACGTTGAAGATTGTTGGTTAGGTGATACTACT
GTATCTTTGCCTGATTTAAACACTACTGAAACTGTCGTAAGGACTATCTGGTATGACTGGGTA
GCTGATTTGGTTTCTAATTACAGTATTGATGGTCTGCGTATAGACACCGTAAAACATGTAGAA
AAATCTTTTTGGCCCGGGTACAACTCTGCAGCGGGTGTCTATTGTGTCGGAGAGGTCTTAGA
TGGGGATCCATCATACACTTGCCCTTATCAGGACTACTTGGATGGTGTCCTAAACTACCCAAT
TTACTACCAATTATTGTATGCCTTCGAATCATCATCTGGGTCAATATCAAATCTTTATAACATG
ATTAATAGCGTAGCCTCAGAATGTTCCGATCCAACACTATTGGGTAATTTCATTGAAAATCAT

GATAACCCGAGATTCGCTAGTTACACTTCTGACTATTCCTTGGCTAAAAACGTGATAGCCTTC
ATTTTTTTTTCCGATGGCATTCCCATTGTTTACGCTGGTCAGGAACAACATTATAATGGTGGT
AACGATCCATATAACAGGGAGGCCACATGGTTATCTGGATACAGCACAACCGCAGAATTGTA
TACTTTCATTGCTACTACAAATGCAATCAGAAGCCTAGCTATATCCGTTGATAGCGAATACTT
GACGTATAAAAATGACCCCTTCTACTACGATTCCAATACATTAGCAATGAGAAAAGGATCTG
ACGGACTGCAAGTGATAACCGTTTTATCTAACTTAGGAGCTGACGGTTCTTCTTATACCTTAA
CATTGTCCGGTTCAGGTTATAGCTCAGGCACAGAGTTAGTGGAGGCTTATACTTGCACCACA
GTTACAGTCGATTCTAATGGTGACATCCCCGTCCCAATGGAGTCAGGCTTACCACGTGTATT
CTTGCCGGCCTCTTCATTTTCTGGATCATCACTGTGTTCTTCTAGCCCATCACCAACAACAAC
TACAAGTACTTCCACCTCTACTACTTCTACAGCATGTACTACTGCTACCGCTGTAGCAGTTTT
ATTTGAAGAGCTGGTTACCACCACTTATGGTGAAAATGTTTACTTGAGTGGCTCAATCTCTC
AGCTAGGCGACTGGAATACTGATGATGCTGTTGCATTGAGCGCTGCCAATTACACATCCAGC
AACCCGTTGTGGTATGTCACAGTTACGTTACCAGTTGGTACCTCATTTGAGTACAAGTTCATT
AAGAAAGAAGAGAATGGCGATGTGGAGTGGGAATCTGATCCAAATAGATCATATACTGTAC
CGACTGCATGTACAGGTGCAACTGAAACAATTGTTGACACTTGGAGATAA

ATGACACCTTTTGTGTTAACAGCAGTTCTATTCTTATTAGGTAATGCTGTACTGGCATTAACG
CCTGCAGAATGGAGAAAGCAGTCTATTTATTTTCTTTTAACCGACAGATTTGGTAGAGCTGA
TAATAGCACTACTGCAGCATGTGATGTCACAGAGCGTATTTATTGTGGTGGCAGTTGGCAGG
GTATCATTAATCACTTGGATTATATCCAAGGGATGGGTTTCACGGCCATTTGGATAAGTCCCG
TGACCGAGCAGCTGCCACAAAACACGGGGGAAGGGGAAGCGTACCACGGGTATTGGCAGC
AGGAAATTTACACCGTAAATTCAAATTTCGGAACTAGTGATGATTTGTTAGCCCTGAGTAAG
GCCTTGCATGATAGAGGGATGTATCTGATGGTGGATGTAGTGGCAAACCATATGGGATACGA
TGGAGATGGAGATTCTGTCGACTATTCCGTTTTCAACCCGTTTAATAGCTCATCCTACTTTCA
TCCCTACTGTCTTATTACAGACTACTCTAACCAGACGGACGTCGAGGATTGTTGGCTAGGAG
ATACTACTGTCAGTCTGCCGGATCTTAACACAACAGAGACTGTAGTCCGTACAATCTGGTAC
GACTGGGTCGCTGACCTGGTATCAAACTACTCTATTGATGGTTTAAGGATTGATACTGTCAA
GCACGTTGAAAAATCATTCTGGCCCGGTTACAATTCCGCCGCAGGCGTATATTGTGTGGGTG
AGGTGTTGGATGGTGACCCGTCATATACGTGTCCGTACCAGGACTATTTGGACGGCGTTCTG
AACTACCCAATCTACTATCAGCTTCTTTACGCTTTTGAATCTTCTTCTGGGAGCATCAGCAAT
CTATATAACATGATCAACAGTGTCGCCTCAGAATGCTCAGACCCAACTCTACTAGGTAATTTC
ATAGAGAATCACGATAACCCCAGGTTTGCTAGTTATACCTCAGATTACTCCCTGGCCAAGAA
TGTTATAGCCTTTATCTTTTTCAGCGATGGAATACCCATCGTTTATGCTGGGCAAGAACAACA
CTATAATGGCGGCAACGACCCCTACAATAGGGAAGCAACTTGGCTAAGTGGTTATTCCACCA
CAGCCGAGTTGTACACGTTTATTGCAACCACAAACGCGATACGTTCTCTGGCCATTAGTGTC
GATTCCGAGTATTTGACCTACAAGAATGACCCATTCTACTATGACAGTAACACTCTTGCAATG
AGAAAAGGTAGCGACGGCCTTCAAGTGATAACTGTGTTAAGCAATCTGGGGGCAGATGGAT
CTAGCTACACACTGACTTTATCTGGATCAGGCTACTCAAGTGGTACTGAGTTAGTAGAGGCT
TACACCTGTACGACGGTTACAGTCGACAGTAACGGCGACATACCTGTACCGATGGAATCAG
GCCTGCCCAGAGTATTTCTTCCCGCTTCCTCCTTCTCAGGAAGTTCACTGTGTTCTTCTAGCC

CCAGCCCCACGACTACCACTAGCACAAGTACATCTACCACCAGTACGGCTTGTACAACAGC
CACAGCTGTTGCCGTTCTATTCGAAGAGCTAGTGACAACCACTTATGGTGAAAACGTGTACC
TGAGTGGGTCTATCAGTCAGCTGGGAGACTGGAACACCGATGATGCCGTCGCTCTGTCTGC
AGCCAACTATACCAGTTCTAACCCCCTTTGGTACGTCACGGTAACCTTACCAGTGGGCACCA
GCTTCGAGTATAAATTCATAAAGAAAGAGGAAAACGGGGATGTCGAGTGGGAATCTGACCC
TAATCGTAGCTATACAGTACCTACCGCATGTACTGGAGCAACAGAGACGATCGTAGACACCT
GGAGATAG

ATGACCCCTTTTGTTCTTACTGCTGTTCTATTTTTGTTGGGTAACGCTGTTTTGGCTTTGACC
CCTGCTGAATGGAGAAAACAATCTATCTATTTTTATTGACCGATAGATTTGGTAGGGCTGAT
AATTCTACAACAGCTGCTTGTGATGTTACTGAAAGAATATATTGTGGTGGTTCCTGGCAGGG
CATTATCAACCATCTGGACTATATCCAAGGTATGGGTTTTACCGCTATTTGGATTTCTCCAGTT
ACTGAGCAATTGCCACAAAATACTGGTGAAGGTGAAGCATACCACGGCTATTGGCAACAAG
AAATTTACACTGTTAATTCAAACTTTGGCACATCTGATGATTTATTGGCATTGTCTAAAGCAT
TACATGACAGAGGTATGTACTTGATGGTTGATGTTGTTGCGAATCATATGGGCTACGATGGTG
ATGGTGACAGTGTTGATTACTCTGTTTTTAACCCATTTAATTCTTCATCATACTTTCATCCTTA
CTGCTTGATCACTGACTACTCTAATCAAACTGACGTTGAAGACTGTTGGTTGGGTGATACTA
CAGTCTCTTTGCCTGATTTGAATACTACAGAAACTGTTGTTAGAACTATCTGGTATGATTGGG
TGGCTGATCTTGTTTCTAATTACTCAATTGATGGTTTAAGAATTGATACTGTTAAACATGTTG
AAAAATCCTTCTGGCCTGGTTATAATTCTGCTGCCGGTGTTTACTGTGTTGGTGAGGTTTTGG
ATGGTGATCCTTCTTATACATGTCCATATCAAGACTACTTGGATGGTGTCTTGAATTACCCAAT
TTACTATCAATTATTATACGCATTTGAGTCCTCTTCGGGATCCATATCTAACTTATACAATATGA
TTAATTCTGTTGCTTCTGAGTGTTCTGATCCAACCTTATTAGGTAATTTTATCGAGAATCATGA
CAACCCAAGATTTGCCTCTTATACATCTGATTATTCATTGGCAAAAAACGTTATTGCTTTTATA
TTCTTCTCAGATGGTATTCCAATCGTCTATGCTGGTCAAGAACAACATTACAATGGTGGTAAT
GATCCATACAATAGAGAAGCTACCTGGTTAAGTGGTTATTCTACAACTGCTGAATTGTACACT
TTTATTGCCACTACTAATGCCATTAGAAGTTTGGCAATCTCTGTGGATTCGGAATATTTAACG
TATAAAAATGACCCTTTCTACTATGATTCTAATACTCTTGCGATGAGAAAGGGTTCAGATGGT
TTGCAAGTTATTACTGTTCTGAGTAATTTAGGTGCTGATGGTTCTTCTTATACCTTGACCTTG
AGTGGTTCTGGGTATTCTTCTGGTACTGAATTGGTTGAAGCTTACACCTGTACAACAGTTAC
CGTAGATTCTAACGGTGATATTCCTGTCCCTATGGAATCTGGTCTTCCAAGAGTTTTTTTACC
AGCTTCTTCTTTTTCTGGTTCTTCTCTATGTTCTTCTTCTCCATCCCCAACTACTACAACTTCC
ACTTCTACCAGCACTACTAGTACAGCTTGTACGACTGCAACAGCTGTCGCTGTTTTATTCGA
AGAATTAGTTACTACCACATACGGCGAAAATGTCTACTTGTCAGGTTCTATTTCACAGTTAGG
TGATTGGAATACTGACGATGCTGTGGCCTTATCTGCTGCTAATTATACTAGCTCTAATCCATTA
TGGTATGTCACTGTTACACTTCCAGTTGGTACTTCTTTCGAATACAAATTCATTAAGAAGGAG
GAGAATGGTGATGTTGAATGGGAATCTGATCCAAATAGGTCTTACACTGTGCCAACTGCTTG
CACTGGTGCTACAGAAACTATCGTCGATACTTGGAGATAA

ATGACGCCTTTCGTCCTCACGGCCGTGCTGTTCTTGCTGGGGAATGCCGTGTTGGCCTTGAC
CCCGGCCGAATGGCGCAAACAATCTATCTACTTTCTCCTCACGGACCGCTTTGGCAGGGCA
GATAACTCGACCACTGCTGCCTGCGATGTCACTGAGAGGATCTACTGTGGCGGGAGTTGGC
AAGGAATCATCAACCATCTCGACTATATCCAAGGCATGGGGTTCACGGCCATCTGGATTTCA
CCGGTGACCGAGCAGCTGCCGCAAAATACGGGTGAGGGAGAAGCCTATCATGGGTATTGGC
AGCAGGAAATATACACGGTCAACTCCAACTTTGGGACATCAGACGATCTCTTAGCCCTGTCA
AAGGCGCTCCATGACCGTGGCATGTACCTCATGGTCGATGTGGTTGCGAATCACATGGGATA
CGATGGAGATGGCGACTCCGTTGATTACAGCGTCTTCAATCCATTTAATTCCTCGAGTTATTT
CCATCCCTATTGCCTGATTACAGACTACAGCAATCAGACCGATGTGGAAGACTGTTGGCTGG
GCGATACGACTGTCTCGTTGCCCGATCTCAACACCACGGAGACTGTTGTGAGGACTATATGG
TATGACTGGGTGGCGGATCTCGTCTCCAATTACTCTATTGATGGGCTTCGCATCGACACGGT
GAAACACGTAGAAAAGTCATTCTGGCCTGGTTACAACAGTGCTGCGGGTGTCTACTGTGTT
GGCGAGGTCCTCGATGGAGATCCGTCTTACACTTGTCCCTACCAGGATTATCTGGACGGTGT
ATTAAACTATCCAATATACTATCAACTACTGTATGCGTTTGAATCCTCTAGCGGCAGCATCAG
CAATCTTTACAACATGATCAACTCTGTCGCCTCTGAATGTTCCGATCCCACTCTGTTGGGCA
ACTTTATCGAGAACCATGACAACCCTAGATTTGCCTCCTATACAAGTGATTATTCTCTTGCTA
AAAATGTGATTGCTTTCATCTTCTTCTCTGACGGCATCCCTATCGTCTATGCCGGTCAGGAGC
AGCATTACAACGGGGGAAATGACCCCTACAACCGCGAGGCCACCTGGCTGTCAGGATACTC
GACGACGGCCGAACTGTACACGTTCATTGCGACCACCAACGCGATCCGTAGCTTGGCGATC
TCCGTCGACTCGGAGTATTTGACGTACAAGAATGACCCATTCTACTACGACAGCAATACCCT
CGCTATGCGCAAGGGTTCGGATGGCCTGCAGGTCATCACTGTTCTGTCCAATCTGGGCGCCG
ATGGTAGCTCGTACACGTTGACTCTGAGTGGCAGTGGCTATTCGTCAGGCACGGAGCTGGT
GGAAGCTTACACCTGCACAACGGTCACTGTTGACTCTAATGGCGATATTCCAGTTCCCATGG
AGTCCGGACTGCCGCGCGTTTTCCTACCAGCATCCTCATTCAGTGGTAGCAGTCTATGCAGT
TCTTCTCCTAGCCCTACTACTACAACATCGACATCGACATCGACAACGTCGACGGCCTGCAC
CACCGCCACCGCTGTGGCGGTCCTCTTCGAAGAGTTGGTGACAACGACCTACGGTGAAAAT
GTCTACCTCAGCGGATCGATCAGCCAACTCGGGGACTGGAACACGGACGACGCCGTGGCC
CTGTCCGCAGCTAATTACACTTCTTCGAATCCCCTGTGGTATGTGACAGTCACATTGCCGGTT
GGGACGTCCTTTGAGTACAAGTTCATCAAGAAGGAAGAGAACGGCGATGTCGAGTGGGAG
AGCGATCCCAATCGGTCGTATACTGTGCCGACGGCCTGCACGGGAGCGACGGAGACGATTG
TCGACACATGGAGATAG

3. **Adalimumab (Humira) Heavy Chain**

GCCCAGCTGGAGGTGCAGCTGGTGGAGAGCGGCGGCGGCCTGGTGCAGCCTGGCAGAAG
CCTGAGACTGAGCTGTGCCGCCAGCGGCTTCACCTTCGACGACTACGCCATGCACTGGGTG
AGACAGGCCCCTGGCAAGGGCCTGGAGTGGGTGAGCGCCATCACCTGGAACAGCGGCCAC
ATCGACTACGCCGACAGCGTGGAGGGCAGATTCACCATCAGCAGAGACAACGCCAAGAAC
AGCCTGTACCTGCAGATGAACAGCCTGAGAGCCGAGGACACCGCCGTGTACTACTGTGCCA
AGGTGAGCTACCTGAGCACCGCCAGCAGCCTGGACTACTGGGGCCAGGGCACCCTGGTGA
CCGTGAGCAGCGCCAGCACCAAAGGTCCCAGCGTGTTCCCTCTGGCCCCTAGCAGCAAGA
GCACCAGCGGCGGCACCGCCGCCCTGGGCTGTCTGGTGAAGGACTACTTCCCTGAGCCTGT
GACCGTGAGCTGGAACAGCGGCGCCCTGACCAGCGGCGTGCACACCTTCCCTGCCGTGCT
GCAGAGCAGCGGCCTGTACAGCCTGAGCAGCGTGGTGACCGTGCCTAGCAGCAGCCTGGG
CACCCAGACCTACATCTGTAACGTGAACCACAAGCCTAGCAACACCAAGGTGGACAAGAA
GGTGGAGCCTAAGAGCTGTGACAAGACCCACACCTGTCCTCCTTGTCCTGCCCCTGAGCTG
CTGGGCGGCCCTAGCGTGTTCCTGTTCCCTCCTAAGCCTAAGGACACCCTGATGATCAGCAG
AACCCCTGAGGTGACCTGTGTGGTGGTGGACGTGAGCCACGAGGACCCTGAGGTGAAGTT
CAACTGGTACGTGGACGGCGTGGAGGTGCACAACGCCAAGACCAAGCCTAGAGAGGAGC
AGTACAACAGCACCTACAGAGTGGTGAGCGTGCTGACCGTGCTGCACCAGGACTGGCTGA
ACGGCAAGGAGTACAAGTGTAAGGTGAGCAACAAGGCCCTGCCTGCCCCTATCGAGAAGA
CCATCAGCAAGGCCAAGGGCCAGCCTAGAGAGCCTCAGGTGTACACCCTGCCTCCTAGCAG
AGACGAGCTGACCAAGAACCAGGTGAGCCTGACCTGTCTGGTGAAGGGCTTCTACCCTAG
CGACATCGCCGTGGAGTGGGAGAGCAACGGCCAGCCTGAGAACAACTACAAGACCACCCC
TCCTGTGCTGGACAGCGACGGCAGCTTCTTCCTGTACAGCAAGCTGACCGTGGACAAGAG
CAGATGGCAGCAGGGCAACGTGTTCAGCTGTAGCGTGATGCACGAGGCCCTGCACAACCA
CTACACCCAGAAGAGCCTGAGCCTGAGCCCTGGCAAGTAG

GCCCAGCTGGAGGTGCAGCTGGTGGAGTCCGGCGGCGGCCTGGTGCAGCCCGGCAGGTCC
CTGAGGCTGTCCTGCGCCGCCTCCGGCTTCACCTTCGACGACTACGCCATGCACTGGGTGA
GGCAGGCCCCCGGCAAGGGCCTGGAGTGGGTGTCCGCCATCACCTGGAACTCCGGCCACA
TCGACTACGCCGACTCCGTGGAGGGCAGGTTCACCATCTCCAGGGACAACGCCAAGAACT
CCCTGTACCTGCAGATGAACTCCCTGAGGGCCGAGGACACCGCCGTGTACTACTGCGCCAA
GGTGTCCTACCTGTCCACCGCCTCCTCCCTGGACTACTGGGGCCAGGGCACCCTGGTGACC
GTGTCCTCCGCCTCCACCAAGGGCCCCTCCGTGTTCCCCCTGGCCCCCTCCTCCAAGTCCAC
CTCCGGCGGCACCGCCGCCCTGGGCTGCCTGGTGAAGGACTACTTCCCCGAGCCCGTGACC
GTGTCCTGGAACTCCGGCGCCCTGACCTCCGGCGTGCACACCTTCCCCGCCGTGCTGCAGT
CCTCCGGCCTGTACTCCCTGTCCTCCGTGGTGACCGTGCCCTCCTCCTCCCTGGGCACCCAG
ACCTACATCTGCAACGTGAACCACAAGCCCTCCAACACCAAGGTGGACAAGAAGGTGGAG
CCCAAGTCCTGCGACAAGACCCACACCTGCCCCCCCTGCCCCGCCCCCGAGCTGCTGGGCG
GCCCCTCCGTGTTCCTGTTCCCCCCCAAGCCCAAGGACACCCTGATGATCTCCAGGACCCC
CGAGGTGACCTGCGTGGTGGTGGACGTGTCCCACGAGGACCCCGAGGTGAAGTTCAACTG

GTACGTGGACGGCGTGGAGGTGCACAACGCCAAGACCAAGCCCAGGGAGGAGCAGTACA
ACTCCACCTACAGGGTGGTGTCCGTGCTGACCGTGCTGCACCAGGACTGGCTGAACGGCA
AGGAGTACAAGTGCAAGGTGTCCAACAAGGCCCTGCCCGCCCCCATCGAGAAGACCATCT
CCAAGGCCAAGGGCCAGCCCAGGGAGCCCCAGGTGTACACCCTGCCCCCCTCCAGGGACG
AGCTGACCAAGAACCAGGTGTCCCTGACCTGCCTGGTGAAGGGCTTCTACCCCTCCGACAT
CGCCGTGGAGTGGGAGTCCAACGGCCAGCCCGAGAACAACTACAAGACCACCCCCCCCGT
GCTGGACTCCGACGGCTCCTTCTTCCTGTACTCCAAGCTGACCGTGGACAAGTCCAGGTGG
CAGCAGGGCAACGTGTTCTCCTGCTCCGTGATGCACGAGGCCCTGCACAACCACTACACCC
AGAAGTCCCTGTCCCTGTCCCCCGGCAAGTGA

GCTCAGCTGGAGGTGCAGCTGGTGGAGAGCGGCGGCGGCCTGGTGCAGCCTGGCAGAAGC
CTGAGACTGAGCTGCGCCGCTAGCGGCTTCACCTTCGACGACTACGCCATGCACTGGGTGA
GACAAGCCCCTGGCAAGGGCCTGGAGTGGGTGAGCGCCATCACCTGGAATAGCGGTCACA
TCGACTACGCCGACAGCGTGGAGGGCAGATTCACCATCAGCAGAGACAACGCAAAGAATT
CCCTGTACCTGCAGATGAACAGCCTGAGAGCCGAGGACACCGCCGTGTACTACTGCGCCAA
GGTGAGCTACCTGAGCACCGCTAGCAGCCTGGACTACTGGGGCCAAGGCACCCTGGTGAC
TGTGAGCTCTGCGAGCACCAAGGGTCCGTCCGTATTCCCTTTGGCCCCTAGCAGCAAGAGC
ACAAGCGGCGGCACCGCCGCCCTGGGCTGCCTCGTTAAGGACTACTTCCCTGAGCCTGTGA
CAGTGTCCTGGAACAGCGGAGCGCTGACAAGCGGCGTGCACACCTTCCCTGCCGTGCTGC
AGAGCAGCGGCCTGTACAGCCTGAGCAGCGTGGTGACCGTGCCTAGCAGCAGCCTGGGCA
CACAGACCTACATCTGCAACGTGAACCACAAGCCTAGCAACACCAAGGTGGACAAGAAGG
TGGAGCCTAAGAGCTGCGACAAGACCCACACCTGCCCTCCTTGCCCTGCCCCTGAGCTGCT
GGGCGGCCCGTCAGTCTTCCTGTTTCCTCCTAAGCCTAAGGACACCCTGATGATCAGCAGA
ACCCCTGAGGTGACCTGCGTGGTGGTGGACGTGAGCCACGAGGACCCTGAGGTGAAGTTC
AACTGGTACGTGGACGGCGTGGAGGTGCACAACGCCAAGACCAAGCCTAGAGAGGAGCA
GTACAACAGCACCTACAGAGTGGTGAGCGTGCTGACCGTGCTGCACCAAGACTGGCTGAA
CGGCAAGGAGTACAAGTGCAAGGTGAGCAACAAGGCCCTGCCTGCCCCTATCGAGAAGAC
CATCAGCAAGGCCAAGGGACAGCCTAGAGAGCCTCAAGTGTACACCCTGCCTCCTAGCAG
AGACGAGCTGACCAAGAACCAAGTGAGCCTGACCTGTTTAGTGAAGGGGTTCTACCCTAG
CGACATCGCCGTGGAGTGGGAGAGCAACGGACAGCCTGAGAACAACTACAAGACCACCCC
TCCTGTGCTGGACAGCGACGGCAGCTTCTTCCTGTACAGCAAGCTGACCGTGGACAAGAG
CAGATGGCAGCAAGGCAACGTGTTCAGCTGCAGCGTGATGCACGAGGCCCTGCACAACCA
CTACACACAGAAGAGCCTGAGCCTGAGCCCTGGCAAGTGA

GCTCAGCTGGAAGTGCAGCTGGTTGAATCTGGCGGAGGACTGGTGCAGCCTGGCAGATCTC
TGAGACTGTCTTGTGCCGCCTCCGGCTTCACCTTCGACGATTACGCTATGCACTGGGTCCGA
CAGGCCCCTGGCAAAGGATTGGAATGGGTGTCCGCCATCACCTGGAACTCCGGCCACATCG
ACTACGCCGATTCCGTGGAAGGCCGGTTCACCATCTCCAGAGACAACGCCAAGAACTCCCT
GTACCTGCAGATGAACAGCCTGAGAGCCGAGGACACCGCCGTGTACTACTGTGCCAAGGT
GTCCTACCTGTCCACCGCCTCTTCTCTGGATTATTGGGGCCAGGGCACCCTGGTCACAGTGT

CCTCTGCTTCTACCAAGGGACCCAGCGTGTTCCCTCTGGCTCCTTCCAGCAAGTCTACCTCT
GGCGGAACAGCTGCTCTGGGCTGCCTGGTCAAGGACTACTTTCCTGAGCCTGTGACCGTGT
CTTGGAACTCTGGCGCTCTGACATCCGGCGTGCACACCTTTCCAGCTGTGCTGCAATCCTCC
GGCCTGTACTCTCTGTCCTCCGTCGTGACCGTGCCTTCTAGCTCTCTGGGCACCCAGACCTA
CATCTGCAATGTGAACCACAAGCCTTCCAACACCAAGGTGGACAAGAAGGTGGAACCCAA
GTCCTGCGACAAGACCCACACCTGTCCTCCATGTCCTGCTCCAGAACTGCTCGGCGGACCT
TCCGTGTTCCTGTTTCCTCCAAAGCCTAAGGACACCCTGATGATCTCTCGGACCCCTGAAGT
GACCTGCGTGGTGGTGGATGTGTCTCACGAGGATCCCGAAGTGAAGTTCAATTGGTACGTG
GACGGCGTCGAGGTGCACAATGCCAAGACCAAGCCTAGAGAGGAACAGTACAACTCCACC
TACAGAGTGGTGTCCGTGCTGACCGTGCTGCATCAGGATTGGCTGAACGGCAAAGAGTACA
AGTGCAAAGTGTCCAACAAGGCCCTGCCTGCTCCTATCGAAAAGACCATCAGCAAGGCCA
AGGGCCAGCCTAGGGAACCCCAGGTTTACACCTTGCCTCCATCTCGGGACGAGCTGACCAA
GAACCAGGTGTCCCTGACCTGCCTCGTGAAGGGCTTCTACCCTTCCGATATCGCCGTGGAAT
GGGAGAGCAATGGCCAGCCAGAGAACAACTACAAGACAACCCCTCCTGTGCTGGACTCCG
ACGGCTCATTCTTCCTGTACTCCAAGCTGACAGTGGACAAGTCCAGATGGCAGCAGGGCAA
CGTGTTCTCCTGCTCCGTGATGCACGAGGCCCTGCACAATCACTACACCCAGAAGTCCCTG
TCTCTGAGCCCCGGCAAATGA

GCACAACTAGAAGTGCAACTAGTGGAAAGTGGCGGCGGCCTGGTGCAGCCTGGCAGATCT
CTGCGGCTGTCCTGTGCTGCTAGCGGCTTCACCTTCGACGACTATGCCATGCACTGGGTGCG
GCAGGCCCCTGGCAAGGGCCTGGAATGGGTCTCCGCCATCACGTGGAATTCCGGCCACATC
GACTATGCCGACTCCGTGGAAGGCAGATTCACAATCAGCCGGGACAACGCCAAGAATTCCC
TGTACCTGCAGATGAACAGTCTCAGGGCCGAGGACACCGCCGTGTACTACTGCGCCAAAGT
GTCCTACCTGTCCACCGCTTCTTCCCTGGATTACTGGGGCCAAGGCACACTGGTTACAGTGT
CTTCTGCTAGCACTAAGGGACCTTCTGTGTTTCCTCTGGCTCCATCCTCTAAGTCTACCTCTG
GCGGAACCGCCGCTCTGGGCTGCCTGGTGAAGGATTACTTCCCCGAGCCTGTGACCGTGTC
ATGGAACTCTGGCGCTCTGACCTCTGGCGTGCACACCTTTCCAGCTGTGCTGCAATCCTCTG
GACTGTACAGCCTGTCCTCCGTCGTGACCGTGCCCAGCTCTTCTCTCGGCACCCAGACCTAC
ATCTGCAACGTGAACCACAAGCCCAGCAACACCAAGGTGGACAAGAAAGTGGAACCTAAA
TCCTGCGACAAGACCCACACCTGTCCTCCTTGCCCTGCCCCTGAACTGCTGGGTGGACCCT
CTGTGTTTCTGTTCCCTCCAAAGCCCAAGGACACACTGATGATCTCTAGAACCCCTGAAGTC
ACCTGCGTGGTGGTGGACGTGTCTCATGAGGATCCTGAGGTGAAGTTCAACTGGTACGTGG
ACGGCGTGGAAGTGCATAATGCCAAAACCAAGCCTCGGGAAGAGCAGTACAACTCCACCT
ACAGAGTGGTGTCCGTGCTGACCGTGTTGCACCAGGACTGGCTGAACGGCAAAGAGTACA
AGTGCAAGGTGTCCAACAAGGCTCTGCCTGCTCCTATCGAGAAGACCATCTCTAAGGCCAA
GGGCCAGCCTAGAGAGCCTCAGGTGTACACCCTGCCTCCTTCCCGCGACGAGCTGACAAA
GAACCAGGTTTCACTGACATGTCTGGTCAAGGGCTTCTACCCCTCCGACATCGCCGTGGAG
TGGGAGTCCAACGGACAGCCTGAGAACAACTACAAGACCACCCCGCCTGTGCTGGACTCT
GATGGCTCCTTCTTCCTGTACTCCAAGCTGACCGTCGATAAGTCCAGATGGCAGCAGGGCA

ACGTGTTCTCCTGCTCCGTGATGCACGAGGCCCTGCACAACCACTACACCCAGAAGAGCCT
GAGTCTGTCTCCCGGCAAATGA

GCTCAACTGGAAGTGCAACTGGTTGAATCCGGCGGCGGCCTTGTACAGCCAGGACGATCCC
TGCGCCTTAGTTGCGCAGCAAGCGGCTTCACTTTCGACGACTATGCAATGCACTGGGTGAG
ACAGGCCCCAGGGAAAGGTCTGGAGTGGGTTAGCGCTATAACTTGGAACAGCGGCCATATC
GATTATGCTGATTCAGTCGAAGGCCGCTTCACCATTTCACGCGACAACGCCAAAAACAGTC
TTTACTTGCAGATGAACAGCCTGAGGGCTGAAGACACAGCCGTGTACTATTGCGCAAAGGT
CAGTTATCTGAGTACAGCTAGCAGCTTGGACTACTGGGGACAAGGAACCCTGGTGACCGTA
TCCAGCGCTAGTACTAAGGGACCTTCCGTATTCCCACTCGCCCCCTCAAGTAAGTCTACTAG
CGGCGGAACAGCAGCCTTGGGTTGTCTGGTCAAAGACTACTTCCCTGAGCCCGTGACCGTG
TCATGGAATTCTGGAGCTCTGACATCAGGGGTGCATACTTTTCCTGCTGTGCTGCAGTCTAG
CGGACTGTACTCCTTGTCATCAGTGGTAACCGTGCCTTCCTCTTCTCTGGGGACTCAGACAT
ACATCTGCAATGTAAACCATAAGCCCAGCAACACTAAGGTGGATAAGAAGGTTGAGCCAAA
GAGCTGCGACAAGACACACACTTGTCCCCCCTGTCCCGCACCCGAACTGTTGGGGGGCCCT
AGCGTGTTTCTCTTCCCCCCTAAGCCAAAGGATACCCTCATGATTAGCCGTACCCCTGAGGT
GACTTGTGTGGTGGTTGATGTTTCTCATGAGGACCCAGAGGTCAAGTTTAACTGGTATGTCG
ATGGAGTGGAGGTACATAATGCAAAGACTAAGCCACGCGAAGAACAGTACAATAGTACTTA
CCGTGTAGTGTCCGTCCTGACTGTACTGCATCAGGACTGGCTGAACGGTAAAGAGTACAAG
TGTAAGGTCAGCAACAAGGCTTTGCCTGCCCCTATCGAGAAGACCATAAGTAAGGCAAAGG
GCCAGCCTCGTGAACCTCAAGTCTACACCCTGCCTCCAAGCCGGGACGAGCTCACTAAGAA
CCAGGTGAGCCTGACTTGTCTGGTAAAGGGCTTCTACCCTTCTGATATCGCCGTGGAATGGG
AGTCTAATGGCCAGCCAGAGAACAATTATAAAACAACCCCACCTGTCCTGGACTCCGATGG
GTCTTTCTTTCTGTACTCCAAGCTCACAGTTGACAAGAGTCGGTGGCAGCAAGGTAACGTG
TTTAGTTGCTCTGTGATGCATGAGGCTCTGCACAACCACTACACCCAAAAGTCACTGTCTCT
GAGCCCTGGGAAATAA

GCGCAACTTGAAGTGCAACTTGTTGAATCAGGGGGAGGTCTTGTCCAGCCCGGCCGCAGTC
TCCGTTTGAGCTGCGCTGCAAGCGGCTTCACATTTGATGACTATGCCATGCATTGGGTACGG
CAGGCTCCCGGCAAAGGACTTGAATGGGTCAGCGCTATAACCTGGAACTCCGGACATATCG
ATTACGCTGATTCTGTCGAAGGCCGGTTTACCATAAGTCGCGACAACGCTAAGAATTCTCTT
TATCTGCAAATGAACTCTCTGCGAGCCGAGGACACTGCAGTTTATTACTGTGCCAAGGTATC
ATATCTCTCTACTGCTTCCAGTTTGGATTATTGGGGACAGGGTACCCTCGTTACTGTCAGTAG
CGCCTCTACTAAAGGCCCAAGTGTCTTTCCTCTGGCTCCATCATCAAAATCTACAAGTGGAG
GTACAGCTGCTCTCGGATGTTTGGTTAAAGACTACTTCCCCGAACCCGTCACTGTTAGCTGG
AACAGTGGAGCACTGACATCAGGCGTTCATACCTTTCCCGCTGTTTTGCAATCCAGTGGCCT
GTATTCTCTCTCTTCTGTTGTCACAGTTCCCTCTTCCTCCTTGGGGACCCAGACATATATATGC
AATGTGAATCATAAACCATCTAATACTAAAGTAGACAAAAAAGTTGAGCCAAAAAGTTGTG
ATAAGACACACACCTGCCCCCCCTGCCCCGCACCAGAGTTGCTTGGTGGACCTTCCGTATTC
CTTTTTCCTCCCAAGCCTAAAGATACTTTGATGATCTCCCGAACTCCTGAAGTCACTTGTGTA

GTCGTTGATGTGTCTCACGAAGATCCAGAGGTCAAATTCAATTGGTACGTGGACGGGGTGG
AGGTCCACAACGCTAAAACTAAGCCTCGCGAGGAACAGTACAACTCCACTTACCGTGTCGT
ATCTGTGCTCACCGTGCTTCATCAGGACTGGCTGAACGGAAAAGAATACAAGTGCAAGGTG
TCAAACAAGGCCCTTCCAGCACCCATCGAGAAAACCATCAGCAAGGCTAAGGGACAGCCC
CGGGAACCCCAAGTGTACACTTTGCCTCCCAGCCGCGATGAGCTTACTAAAAACCAGGTTT
CATTGACCTGTTTGGTAAAAGGCTTTTATCCATCAGACATCGCCGTCGAGTGGGAGAGTAAC
GGACAACCAGAAAACAACTACAAAACAACACCCCCCGTACTGGACAGCGACGGCTCTTTC
TTTCTGTACTCTAAACTCACTGTAGATAAAAGTCGTTGGCAACAAGGTAACGTATTCAGCTG
TAGCGTGATGCATGAAGCCCTGCATAATCATTACACCCAGAAGTCCTTGTCACTCTCACCAG
GAAAATAG

GCCCAGCTGGAGGTGCAGCTGGTGGAGAGCGGCGGCGGCCTGGTCCAGCCAGGGAGAAG
CCTGCGGCTGTCTTGTGCTGCATCAGGCTTTACCTTCGATGATTACGCTATGCATTGGGTGCG
GCAGGCTCCCGGAAAAGGTCTGGAGTGGGTGTCTGCCATTACCTGGAACAGCGGCCACATC
GACTATGCTGACTCTGTGGAGGGCCGGTTCACCATCAGCCGGGACAACGCTAAGAATAGCC
TGTATCTGCAGATGAACAGCCTTCGCGCTGAGGACACCGCTGTGTACTACTGCGCTAAGGTC
AGCTACCTCTCTACCGCCTCCTCCCTGGACTACTGGGGACAGGGTACTCTGGTGACCGTGA
GCTCAGCCTCCACTAAGGGCCCTTCTGTCTTCCCACTGGCCCCTTCTAGCAAGTCTACATCC
GGCGGCACAGCTGCCCTGGGCTGCCTGGTGAAGGACTACTTCCCTGAACCAGTGACCGTG
AGTTGGAACTCCGGCGCCCTGACCTCCGGAGTGCACACCTTCCCTGCCGTGCTGCAGTCCT
CTGGACTGTATAGCCTGTCCTCCGTGGTGACCGTGCCTTCCTCCAGCCTGGGGACACAGAC
TTACATCTGCAACGTGAACCATAAGCCCTCTAACACAAAGGTGGACAAGAAGGTGGAACCT
AAGTCTTGCGATAAGACTCACACCTGCCCTCCTTGTCCCGCCCCAGAGCTGCTGGGAGGCC
CATCCGTCTTTCTGTTCCCACCTAAGCCAAAGGACACACTGATGATTTCTCGGACTCCTGAG
GTGACCTGTGTGGTGGTGGACGTGTCCCACGAGGATCCTGAAGTGAAGTTCAATTGGTACG
TGGATGGCGTGGAGGTGCACAACGCCAAGACCAAGCCTAGAGAGGAGCAGTACAACTCCA
CTTATAGAGTGGTGAGTGTGCTGACCGTTCTGCACCAGGACTGGCTGAATGGTAAGGAGTA
CAAGTGCAAGGTGTCAAACAAGGCACTCCCCGCTCCAATTGAGAAAACAATCTCTAAGGC
CAAGGGCCAGCCTCGGGAACCTCAGGTGTACACCCTGCCCCCCTCTAGGGATGAGTTGACC
AAGAACCAGGTGTCTCTGACCTGTCTGGTGAAGGGCTTTTATCCCTCTGATATCGCCGTGGA
GTGGGAGTCTAACGGACAGCCCGAGAACAACTACAAGACCACCCCACCCGTGCTCGACAG
CGACGGCTCTTTCTTCCTGTACTCCAAATTGACAGTGGACAAGTCTCGGTGGCAGCAGGGA
AACGTGTTCTCTTGCTCCGTGATGCACGAAGCTCTGCATAATCACTACACCCAGAAGTCTCT
GAGCCTGAGCCCTGGCAAGTGA

GCGCAGCTTGAAGTGCAATTGGTTGAATCAGGAGGAGGACTCGTGCAACCGGGAAGGAGT
TTACGATTATCTTGTGCTGCCTCTGGATTCACCTTTGACGACTATGCAATGCATTGGGTCCGT
CAAGCACCAGGAAAAGGTTTAGAGTGGGTTTCAGCAATCACTTGGAACTCCGGACATATTG
ACTATGCCGATAGTGTTGAGGGTCGATTCACAATCTCACGAGATAACGCGAAGAATAGTCTA
TACCTACAGATGAATAGCCTAAGAGCTGAGGATACTGCCGTTTATTACTGTGCAAAGGTTTC

CTATCTTTCTACTGCATCTAGTCTTGATTACTGGGGACAAGGAACACTTGTCACAGTTTCCTC
TGCTAGCACAAAAGGACCTAGCGTTTTCCCTCTGGCACCATCAAGTAAGAGCACCAGTGGC
GGGACAGCAGCACTGGGTTGTCTTGTGAAAGACTATTTCCCAGAACCCGTTACCGTTAGTT
GGAACTCAGGCGCACTTACTTCGGGAGTTCATACTTTTCCTGCTGTCTTACAATCTTCCGGT
CTCTATTCACTAAGCTCAGTTGTCACTGTACCTTCCTCAAGCCTTGGGACACAAACCTACAT
TTGTAACGTCAATCATAAACCGAGCAATACGAAGGTAGATAAGAAAGTCGAGCCAAAGAGT
TGTGATAAAACACACACTTGCCCACCTTGCCCAGCTCCTGAACTCTTAGGTGGACCAAGCG
TTTTCCTCTTTCCTCCAAAGCCGAAAGATACACTTATGATATCACGCACACCCGAAGTTACTT
GTGTGGTTGTAGACGTTTCTCATGAAGATCCCGAAGTGAAGTTTAATTGGTACGTCGATGGT
GTTGAAGTTCACAATGCTAAGACTAAGCCAAGAGAAGAGCAATACAACTCAACCTATAGAG
TTGTTTCCGTCTTAACCGTACTGCATCAAGATTGGTTGAACGGCAAGGAGTATAAATGCAAG
GTTAGCAATAAAGCACTACCTGCACCGATTGAGAAGACAATTAGCAAAGCAAAAGGACAA
CCAAGGGAACCACAAGTCTATACACTTCCACCTTCAAGGGATGAGCTGACTAAGAATCAAG
TATCCTTGACCTGTTTAGTCAAGGGGTTTTACCCTTCTGACATTGCCGTAGAATGGGAATCTA
ATGGGCAGCCTGAGAATAACTATAAGACAACTCCACCCGTACTCGATTCTGACGGCTCTTTT
TTCCTATACTCCAAGCTAACCGTGGATAAATCACGTTGGCAACAAGGAAACGTTTTCTCTTG
TTCTGTGATGCACGAGGCTTTGCATAATCACTACACACAAAAGAGCTTAAGTCTTAGCCCTG
GGAAATAG

**4. Adalimumab (Humira) Light Chain**

GACATCCAGATGACCCAGAGCCCTAGCAGCCTGAGCGCCAGCGTGGGCGACAGAGTGACC
ATCACCTGTAGAGCCAGCCAGGGCATCAGAAACTACCTGGCCTGGTACCAGCAGAAGCCTG
GCAAGGCCCCTAAGCTGCTGATCTACGCCGCCAGCACCCTGCAGAGCGGCGTGCCTAGCAG
ATTCAGCGGCAGCGGCAGCGGCACCGACTTCACCCTGACCATCAGCAGCCTGCAGCCTGA
GGACGTGGCCACCTACTACTGTCAGAGATACAACAGAGCCCCTTACACCTTCGGCCAGGGC
ACCAAGGTGGAGATCAAGAGAACCGTGGCCGCCCCTAGCGTGTTCATCTTCCCTCCTAGCG
ACGAGCAGCTGAAGAGCGGCACCGCCAGCGTGGTGTGTCTGCTGAACAACTTCTACCCTA
GAGAGGCCAAGGTGCAGTGGAAGGTGGACAACGCCCTGCAGAGCGGCAACAGCCAGGAG
AGCGTGACCGAGCAGGACAGCAAGGACAGCACCTACAGCCTGAGCAGCACCCTGACCCTG
AGCAAGGCCGACTACGAGAAGCACAAGGTGTACGCCTGTGAGGTGACCCACCAGGGCCTG
AGCAGCCCTGTGACCAAGAGCTTCAACAGAGGCGAGTGTTGA

GACATCCAGATGACCCAGTCCCCCTCCTCCCTGTCCGCCTCCGTGGGCGACAGGGTGACCA
TCACCTGCAGGGCCTCCCAGGGCATCAGGAACTACCTGGCCTGGTACCAGCAGAAGCCCG
GCAAGGCCCCCAAGCTGCTGATCTACGCCGCCTCCACCCTGCAGTCCGGCGTGCCCTCCAG
GTTCTCCGGCTCCGGCTCCGGCACCGACTTCACCCTGACCATCTCCTCCCTGCAGCCCGAG
GACGTGGCCACCTACTACTGCCAGAGGTACAACAGGGCCCCCTACACCTTCGGCCAGGGCA
CCAAGGTGGAGATCAAGAGGACCGTGGCCGCCCCCTCCGTGTTCATCTTCCCCCCCTCCGA
CGAGCAGCTGAAGTCCGGCACCGCCTCCGTGGTGTGCCTGCTGAACAACTTCTACCCCAGG
GAGGCCAAGGTGCAGTGGAAGGTGGACAACGCCCTGCAGTCCGGCAACTCCCAGGAGTCC
GTGACCGAGCAGGACTCCAAGGACTCCACCTACTCCCTGTCCTCCACCCTGACCCTGTCCA
AGGCCGACTACGAGAAGCACAAGGTGTACGCCTGCGAGGTGACCCACCAGGGCCTGTCCT
CCCCCGTGACCAAGTCCTTCAACAGGGGCGAGTGCTGA

GACATTCAGATGACACAGAGCCCTAGCAGCCTGAGCGCTAGCGTGGGCGACAGAGTGACC
ATCACCTGCAGAGCTAGCCAAGGCATCAGAAACTACCTGGCCTGGTATCAGCAGAAGCCTG
GCAAGGCCCCTAAGCTGCTGATCTACGCCGCTAGCACGCTGCAGAGTGGAGTGCCTAGCAG
ATTCAGCGGCAGCGGCAGCGGCACCGACTTCACCCTGACCATCAGCAGCCTGCAGCCTGA
GGACGTGGCCACCTACTACTGTCAGAGATACAACAGAGCCCCTTACACCTTCGGCCAAGGC
ACCAAGGTGGAGATCAAGAGAACCGTGGCCGCCCCTAGCGTGTTCATCTTCCCTCCTAGCG
ACGAGCAGCTGAAGAGCGGCACCGCTAGCGTGGTGTGCCTGCTGAACAACTTCTACCCTAG
AGAGGCCAAGGTGCAGTGGAAGGTGGACAACGCCCTGCAGAGTGGTAATTCCCAAGAGAG
CGTGACCGAGCAAGACAGCAAGGACAGCACCTACAGCCTGAGCAGCACCCTGACCCTGAG
CAAGGCCGACTACGAGAAGCACAAGGTGTACGCCTGCGAGGTGACCCACCAAGGCCTGAG
CAGCCCTGTGACCAAGAGCTTCAACAGAGGCGAGTGCTGA

GACATCCAGATGACCCAGTCTCCATCCTCTCTGTCCGCCTCTGTGGGCGACAGAGTGACCAT
CACCTGTAGAGCCAGCCAGGGCATCAGAAACTACCTGGCCTGGTATCAGCAGAAGCCCGGC
AAGGCTCCTAAGCTGCTGATCTACGCTGCTAGCACCCTGCAGTCTGGCGTGCCCTCTAGATT
TTCCGGCTCTGGCTCTGGCACCGACTTTACCCTGACAATCTCCAGCCTGCAGCCTGAGGATG
TGGCCACCTACTACTGCCAGCGGTACAACAGAGCCCCTTACACCTTTGGCCAGGGCACCAA
GGTGGAAATCAAGCGGACAGTGGCCGCTCCTTCCGTGTTCATCTTCCCACCTTCCGACGAG
CAGCTGAAGTCCGGCACAGCTTCTGTCGTGTGCCTGCTGAACAACTTCTACCCTCGGGAAG
CCAAGGTGCAGTGGAAGGTGGACAACGCTCTGCAGTCCGGCAACTCCCAAGAGTCTGTGA
CCGAGCAGGACTCCAAGGACAGCACCTACAGCCTGTCCTCCACACTGACCCTGTCCAAGG
CCGACTACGAGAAGCACAAGGTGTACGCCTGCGAAGTGACCCATCAGGGCCTGTCTAGCCC
TGTGACCAAGTCTTTCAACCGGGGCGAGTGCTGA

GATATCCAAATGACTCAAAGTCCAAGTAGTCTCAGCGCCTCCGTGGGCGACAGAGTGACCA
TCACCTGTAGAGCTAGCCAGGGCATCCGGAATTACCTGGCCTGGTACCAGCAGAAGCCTGG
CAAGGCTCCTAAGCTGCTGATCTACGCTGCCTCTACACTGCAATCTGGCGTGCCTTCTCGGT
TCTCCGGCTCTGGCTCCGGCACCGACTTCACCCTGACCATCTCCTCTCTGCAGCCTGAGGAC
GTGGCCACCTACTACTGCCAGAGATACAACCGGGCTCCTTACACCTTCGGCCAGGGCACAA
AAGTGGAAATCAAGCGGACCGTGGCTGCCCCCTCCGTGTTCATCTTTCCTCCTTCCGATGAA
CAGCTGAAATCTGGAACCGCCTCTGTGGTGTGCCTGCTGAACAACTTCTACCCCAGAGAAG
CCAAGGTGCAGTGGAAGGTGGACAACGCCCTGCAGTCCGGCAACTCCCAAGAGTCCGTCA
CCGAGCAGGATAGCAAGGACTCCACCTATTCCCTGTCTTCTACCCTGACCCTGTCCAAGGCC
GACTACGAGAAGCACAAGGTGTACGCTTGCGAGGTGACCCACCAGGGACTGAGCTCTCCA
GTCACAAAGTCCTTCAACAGAGGCGAGTGCTGA

GACATCCAAATGACCCAGAGCCCCTCATCCCTGTCCGCAAGTGTTGGCGACAGAGTAACCA
TAACTTGTAGGGCTTCACAGGGAATCCGCAATTACTTGGCCTGGTATCAGCAGAAACCAGG
TAAGGCCCCAAAGCTGTTGATCTACGCAGCCTCTACCCTTCAGTCTGGCGTACCAAGCCGTT
TCAGCGGCTCTGGAAGTGGAACTGACTTCACCCTGACTATCAGTTCCTTGCAACCCGAAGA
CGTCGCCACATATTATTGTCAGCGGTATAACCGGGCACCATATACCTTCGGTCAGGGTACCAA
GGTGGAGATCAAGAGGACAGTGGCTGCTCCTAGTGTATTCATCTTCCCCCCTAGTGACGAA
CAACTTAAGTCTGGCACCGCTTCTGTGGTCTGCCTGCTCAACAACTTTTACCCCAGGGAGG
CAAAGGTGCAGTGGAAGGTTGACAACGCATTGCAGTCCGGCAACTCTCAAGAGTCCGTGA
CCGAGCAAGACAGCAAGGACAGCACCTACTCTCTCTCTTCCACCCTCACCCTGAGCAAAGC
CGACTATGAGAAACACAAGGTCTACGCTTGTGAGGTGACTCATCAGGGCCTGTCCAGTCCA
GTGACTAAAAGTTTCAACCGTGGAGAGTGTTAA

GACATACAGATGACCCAGAGTCCATCCTCACTTTCTGCAAGCGTAGGAGACAGAGTCACCA
TCACTTGCCGAGCCAGTCAGGGTATCAGAAATTATCTGGCTTGGTACCAGCAGAAGCCTGG
TAAGGCTCCTAAGCTCCTGATCTATGCCGCCAGTACACTTCAGAGCGGAGTCCCTAGCCGGT
TTAGCGGCTCCGGTTCAGGGACCGATTTTACCCTTACTATCTCATCCCTTCAACCTGAGGATG

TAGCCACCTATTACTGCCAGCGTTATAACCGAGCACCTTATACTTTTGGCCAGGGAACAAAG
GTGGAGATAAAACGAACTGTGGCCGCTCCATCAGTGTTCATATTCCCACCTAGCGATGAGCA
GTTGAAAAGCGGTACCGCCTCCGTTGTTTGCCTGCTTAATAATTTTTACCCACGCGAGGCCA
AAGTTCAGTGGAAAGTCGACAACGCACTTCAAAGCGGAAATAGCCAAGAGTCAGTCACTG
AACAGGATAGTAAAGACAGCACCTATAGCCTCTCAAGTACCCTCACCTTGAGCAAGGCCGA
CTACGAAAAACACAAAGTTTATGCTTGCGAAGTAACCCACCAGGGCCTTAGTTCCCCTGTA
ACTAAGTCCTTCAATCGAGGCGAATGCTGA

GACATCCAGATGACACAGTCTCCTAGCTCTCTGAGCGCCAGTGTGGGCGACCGAGTGACCA
TCACCTGTAGGGCTTCTCAGGGCATCCGGAACTACCTGGCATGGTACCAGCAGAAGCCAGG
CAAGGCCCCCAAGCTGCTGATCTACGCCGCTTCCACCCTGCAGTCCGGGGTCCCCAGCCGG
TTCTCTGGTTCTGGGAGCGGCACCGACTTTACACTGACTATTAGTAGCCTGCAGCCCGAGGA
CGTGGCCACCTACTATTGCCAGAGGTACAACAGGGCTCCTTACACCTTCGGTCAGGGCACA
AAGGTGGAGATCAAGAGAACCGTGGCCGCCCCTTCCGTGTTTATCTTTCCACCATCTGACG
AGCAGCTCAAGTCCGGCACCGCTAGCGTGGTGTGCCTGCTGAATAACTTCTACCCCCGGGA
GGCTAAGGTGCAGTGGAAGGTGGACAACGCTCTGCAGAGTGGCAACAGTCAGGAGTCCGT
GACCGAGCAGGACTCAAAGGACAGCACTTACTCTCTGTCCTCCACCCTGACACTGTCCAAG
GCTGACTACGAGAAGCACAAAGTGTACGCCTGTGAGGTGACCCACCAGGGCCTGTCAAGC
CCCGTGACTAAGTCCTTTAACAGGGGAGAATGCTGA

GACATACAAATGACTCAATCTCCAAGTTCACTATCTGCTTCAGTCGGCGATAGGGTCACTAT
AACTTGTAGAGCCTCTCAGGGCATAAGAAACTATTTGGCATGGTACCAACAGAAACCTGGA
AAAGCTCCTAAGCTGCTAATATATGCTGCTTCTACACTTCAGAGTGGAGTACCTTCAAGATT
CAGTGGATCTGGTTCTGGGACTGATTTCACTTTGACTATCTCATCCCTCCAACCAGAAGACG
TTGCTACATACTATTGCCAGCGCTATAATAGGGCTCCTTATACCTTTGGACAAGGCACAAAAG
TCGAGATTAAGAGAACTGTTGCTGCACCATCAGTGTTTATTTTCCCTCCAAGTGACGAACAG
CTTAAATCTGGAACTGCAAGCGTTGTATGCCTTCTCAACAATTTCTACCCTAGAGAAGCGAA
AGTCCAATGGAAAGTAGATAACGCACTTCAGTCTGGGAACTCACAAGAGAGTGTCACTGA
ACAAGATTCGAAAGACTCTACCTATTCACTCTCATCGACTCTTACTCTGTCAAAAGCTGATT
ACGAGAAGCACAAAGTGTATGCTTGCGAAGTTACACACCAAGGACTTAGCTCACCAGTAAC
CAAGAGCTTCAATAGGGGAGAATGCTGA
